# Estimating the environmental burden of disease in children and adolescents in Germany: limitations and possible solutions

**DOI:** 10.3389/fpubh.2026.1796283

**Published:** 2026-04-10

**Authors:** Myriam Tobollik, Sarah Kienzler, Dirk Wintermeyer, Dietrich Plass

**Affiliations:** 1 Section Environmental Medicine and Health Effects Assessment, German Environment Agency, Berlin, Germany; 2 Section Exposure Assessment and Environmental Health Indicators, German Environment Agency, Berlin, Germany

**Keywords:** adolescents, children, comparative risk assessment, environmental burden of disease, GerES V, German Environmental Survey, Germany

## Abstract

**Introduction:**

Children and adolescents play an exceptional role in our society especially when it comes to safeguarding their health and given their higher susceptibility to environmental risk factors and ongoing physical development.

**Method:**

We used data from the population-representative cross-sectional German Environmental Survey for Children and Adolescents (GerES V) and assessed the feasibility of applying the environmental burden of disease method for estimating the burden of environmental risks for children and adolescents in Germany. In this context, we highlight the challenges and discuss potential strategies how to deal with them.

**Results:**

In a four steps approach encompassing exposure assessment, systematic literature search, input data search and environmental burden of disease quantification, we aimed to estimate the impact of 17 risk factors on the health of children and adolescents. Following exposure assessment, eight risk factors were excluded. For the remaining risk factors systematic literature searches were conducted, which yielded exposure-response functions for five risk factors. The subsequent burden of disease estimations for benzene, bisphenol A, particulate matter, secondhand smoke and traffic noise were not fully standardized, allowing only limited comparability.

**Discussion:**

In the end, the burden of disease calculations performed varied widely for each risk factor. This variance can be attributed to diverse factors along the calculation processes, including, e.g., the absence of exposure-response functions tailored to children, insufficient exposure data, and gaps in other input data.

## 1 Introduction

A healthy and in the best-case pollutant free environment is necessary for a healthy development of children and adolescents. Children are generally a more vulnerable group compared to adults. Health impairments resulting from different risks in early life can affect the development of many organ systems and lead to severe health problems in later life, and in the worst case, affect the entire life span (1). For instance, children breath more air in relation to their body weight than adults. Their lungs are still growing, making them more susceptible to harmful effects of air pollutants. This can lead to irreversible damage and impaired lung function (2).

To better understand the state of the children’s environment and its impacts on their health, the German Environment Agency conducted the German Environmental Survey for Children and adolescents (GerES V). In this population-representative cross-sectional study the environmental exposure of children and adolescents living in Germany was assessed (3). Therefore, a range of substances was measured in the blood and urine of the participants, in indoor and outdoor air, household dust, drinking water, and several characteristics of both behaviors and living conditions were assessed by questionnaires.

An approach to quantify the impact of environmental risks on human health is the environmental burden of disease (EBD) method, developed by the World Health Organization (WHO) (4). The EBD methodology builds upon the Comparative Risk Assessment concept where the burden of disease attributable to various risk factors is calculated in a comprehensive, consistent and thus comparable way (5). The EBD method was selected for this study due to its ability to provide comprehensive, comparable, and policy-relevant estimates of health impacts resulting from environmental exposures. Unlike epidemiological cohort studies which often focus on specific populations or single exposures, EBD combines data from multiple sources to quantify health outcomes, e.g., in terms of disability-adjusted life years (DALYs) on population level (e.g., country). This approach enables comparisons across different environmental risks and beyond to support priority setting in (environmental) public health. EBD offers a standardized tool for assessing environmental health impacts, particularly in contexts where informing policy and prevention strategies is a primary goal.

A series of reports by the European Topic Centre on Human Health and the Environment have documented a wide range of health impacts among children and adolescents in Europe associated with exposure to air pollution, hazardous chemicals, noise, and climate change-related factors (6–9). These findings are further supported by a growing body of studies providing quantitative estimates of the disease burden attributable to environmental exposures, underscoring the importance of addressing environmental determinants of child health in Europe. A study by Valent et al. (10) estimated that among European children aged 0 to 4 years, outdoor air pollution accounted for 2 to 6% of all-cause deaths, while indoor air pollution-related acute lower respiratory infections caused 5% of deaths. Mild mental retardation due to lead exposure contributed to 4% of DALYs in this age group. For children aged 0 to 14 years, diarrhea linked to inadequate water and sanitation was responsible for 5% of deaths, and injuries accounted for 23% of deaths among those aged 0 to 19 years (10). The authors also reported substantial uncertainties around some of the estimates, particularly for outdoor air pollution, due to limitations in data quality and methodological challenges. More recently Rojas-Rueda and colleagues estimated that children under 14 years of age lost about 13,000 years of life due to exposure to dampness and mold, 33,000 years of life due to secondhand smoke, and about 6,200 years of life due to lead exposure in the year 2015 (11). A study focusing on secondhand smoke exposure in children and during pregnancy across Europe estimated that in 2017, 335 deaths and 35,633 DALYs in children were attributable to secondhand smoke with low birth weight as the leading contributor to disease burden (12). In addition, chemicals, especially endocrine disrupting components, pose a risk to children’s health (13, 14). For many chemicals, however, estimates of the population health impact are not available.

Information on the EBD is increasingly used to inform policy-making. One illustrative example is the application of this method in the revision of the European Clean Air Directive (15). Another illustrative example is the annually updated report published by the European Environment Agency on the burden of disease caused by various air pollutants in Europe (16, 17). In the context of burden of disease, the Global Burden of Disease (GBD) study is a particularly outstanding example of an extensive and comprehensive analysis. The study, which encompasses calculations for 88 risk factors, with 11 from the environmental field, represents a cornerstone for such assessments as it provides the most comprehensive global analyses (18). Despite the GBD-study and a few national assessments of the EBD, studies focusing on environmental risk factors and the population group of children and adolescents are scarce. Especially studies focussing on risks resulting from chemicals in the environment are necessary but lacking.

The main objective of the UKAGEP (“Umweltbedingte Krankheitslasten und Gesundheitliche Parameter”; Environmental Burden of Disease and Health Parameters) project was to test the feasibility of using the EBD methodology to estimate the burden of disease associated with the exposure to environmental risk factors of children and adolescents living in Germany (19). In this article we focus on the challenges encountered when quantifying EBD for children and adolescents in Germany (Table 1). The GerES V study served us as a valuable input data source but also revealed some additional challenges. Our aim is to explain the advantages and the difficulties associated with the use of such data.

**Table 1:** Objective of the article and considered environmental risk factors.

Objective of the article
Explaining the advantages and challenges when quantifying the burden of disease for 17 environmental risk factors impacting children and adolescents in Germany
Considered environmental risk factors
Arsenic Benzene Bisphenol A (BPA) Cadmium Chromium VI Formaldehyde Lead Naphthalene Particulate matter (PM_2.5_, PM_10_) Perfluorinated organic compounds (PFC) Phthalates Polychlorinated biphenyls (PCB) Secondhand smoke Terpenes Traffic noise TVOC (Total Volatile Organic Compounds) as the sum of volatile organic compounds in indoor air Ultrafine particles (PM_0.1_)

## 2 Methods

The aim of the article is to describe our approach for estimating the EBD of children and adolescents aged 3 to 17 years, living in Germany. Additionally, we will delineate the limitations and challenges encountered during the process and propose potential solutions.

Our approach follows four steps, which are explained in detail below. However, as a preliminary step, we first describe the selection process which led us to the considered risk factors.

### 2.1 Preliminary step: risk factor pre-selection

The selection of risk factors is an essential prerequisite to the calculation of the EBD. The selection was guided by one main criterion: We aimed to start as inclusive as possible, considering a large number of risks that are relevant to public health and may pose potential harm to human health, even if EBD quantifications were not yet available for all of them. Thus, inclusion was based on the general existence of scientific evidence linking these risk factors to measurable adverse health effects. Our selection was primarily informed by results of previous studies such as GerES IV, which assessed the concentrations of most of these risk factors in different media, highlighting their relevance for children and adolescents in Germany (20). For the cancerogenic risk factors, this potential health effects have been confirmed through numerous individual studies as well as comprehensive reports from international organizations such as the WHO and Agency for Toxic Substances and Disease Registry. In contrast, for other risk factors such as ultrafine particles, the body of evidence is limited, though current studies suggest potential harmful effects.

To be as thorough as possible, we chose to begin with an extensive list of risk factors. At this stage, no systematic search was performed. The selected environmental risk factors were as follows:

- Arsenic
- Benzene
- Bisphenol A (BPA)
- Cadmium
- Chromium VI
- Formaldehyde
- Lead
- Naphthalene
- Particulate matter (PM_2.5_, PM_10_)
- Perfluorinated organic compounds (PFC)
- Phthalates
- Polychlorinated biphenyls (PCB)
- Secondhand smoke
- Terpenes
- Traffic noise
- Total Volatile Organic Compounds (TVOC) as the sum of volatile organic compounds in indoor air
- Ultrafine particles (PM_0.1_)

### 2.2 First step: exposure assessment

For the 17 risk factors selected in the preliminary step, exposure data from the GerES V study was obtained to determine whether the level of exposure could possibly have an adverse effect on health and thus potentially cause a considerable disease burden. The GerES V study provided data from human biomonitoring (concentration of substances in human tissues such as urine and blood samples) as well as concentrations of pollutants in drinking water, household dust and indoor air samples. Additionally, questionnaires were used to assess individual characteristics, relevant behaviors and information about living conditions. The sample comprised 2,294 children and adolescents aged 3 to 17 years living in Germany. It was a population-representative cross-sectional study, with the field work conducted between 2014 and 2017. Further details can be found in the publication by Schulz et al. (3).

The GerES V study was conducted as the environmental module of the German Health Interview and Examination Survey for Children and Adolescents (KiGGS 2), which was carried out by the Robert Koch Institute (21). The data collected in KiGGS 2 include objective measurements of physical and mental health, along with parent- or self-reported information on subjective health status, health behaviors, healthcare utilization, social and migration status and living conditions.

### 2.3 Second step: systematic literature search and selection of risk estimates

In a second step, we conducted systematic literature searches to identify exposure-response functions (ERFs) for the selected risk-health outcome pairs. The selection of the risk-health outcome pairs was guided by the expertise of professionals in the field and by consulting reviews from established institutions. The three databases PubMed, Scopus and LIVIVO were used for the systematic literature search. Search terms were formulated with the assistance of a librarian, combining the risk factors identified in step 1 with health effects associated with the exposure.

The search methodology followed the recommendations of the Cochrane Manual on systematic literature reviews (22). A multi-stage procedure was performed. After the identification of articles by applying the search strings in the selected search engines, all entries were transferred to a database file specific to each risk factor. After removing duplicates, the articles were screened independently by two researchers. The process involved first reading the title, then the abstract, and finally the full-text. Predefined inclusion and exclusion criteria were applied for the selection of relevant articles. For each risk factor, a Preferred Reporting Items for Systematic Reviews and Meta-Analyses (PRISMA) statement was formulated (23). The quality of the studies was assessed using the assessment tool of the National Health, Lung and Blood Institute (24). The studies were rated as good, fair or poor, based on a qualitative overall judgment rather than a numerical score. For secondhand smoke an umbrella review was conducted with focus on already available systematic reviews. Here the quality assessment tool AMSTAR 2 (A measurement tool to assess systematic reviews) was used in an adapted form (25). Only articles with at least a “good” quality rating were included in the selection of ERFs. To ensure the reliability of the results, we decided to consider only those health outcomes for which at least three single studies were available. For more details on the literature search please see the Supplementary material.

### 2.4 Third step: input data

In a third step, the input data for burden of disease quantifications were collected. These were:

- Population data stratified by age and sex
- Life expectancy at age of death stratified by age and sex
- Number of deaths stratified by age and sex (health outcome specific)
- Prevalence data stratified by age and sex (health outcome specific)
- Disability Weights stratified by age and sex (health outcome specific)
- Exposure-response function (identified in step 2) (health outcome and risk factor specific)
- Exposure data stratified by age and sex (identified in step 1)

### 2.5 Fourth step: environmental burden of disease quantification

In the final step, the EBD was quantified using the EBD approach introduced by the WHO (4). The EBD approach is based on the general idea of the Comparative Risk Assessment using the core indicator, DALY, which comprises the years of life lost due to premature mortality (YLL) and the years lived with disability (YLD) (5). The DALYs are the sum of YLLs and YLDs. YLLs are calculated by multiplying the number of sex- and disease-specific deaths in an age group by the remaining life expectancy at age of death. YLDs are determined by multiplying the number of prevalent cases of a disease in a given year by a weighting factor (disability weight) that reflects the impact of the disease on health, using a scale where 0 equals full health and 1 equals death. One DALY represents the loss of 1 year of healthy life.

The core idea of the EBD concept and the Comparative Risk Assessment is the comparison of an observed exposure against a hypothetical exposure, the so-called counterfactual exposure. The disease burden is then estimated as the difference in the health outcomes between these two exposures. To determine the portion of the disease burden attributable to the specific risk factor, the total disease burden for a given health outcome is multiplied by the population attributable fraction (PAF) (4). The PAF was calculated with this equation (26):


$$\mathrm{PAF}=\frac{\sum_{i=1}^{n}P_{1}(RR_{i}-1)}{\sum_{i}^{n}P_{1}(RR_{i}-1)+1}$$

To estimate the PAF, we need the Relative Risk (RR) for a specific health outcome associated with exposure to a risk factor. The RR quantifies the strength of the association between the risk factor and the health outcome. By combining this with information on the proportion of the population exposed to the risk factor (P in the equation above), we can determine what percentage of the total disease burden is attributable to that exposure.

## 3 Results

**Figure 1:**
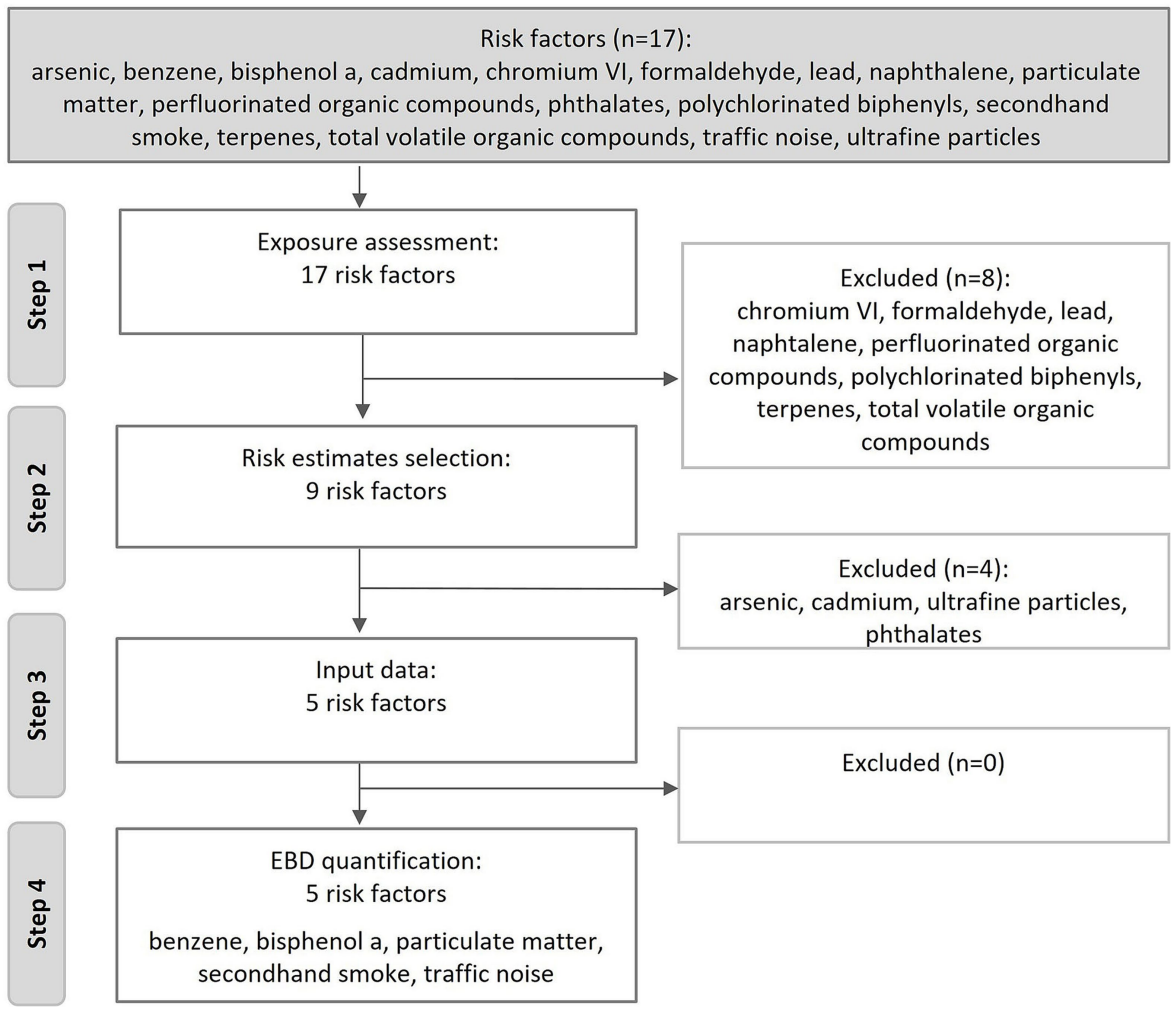
Flow diagram of the four work steps with the risk factors and at which steps they were excluded.

Our aim was to estimate the burden of disease of the 17 risk factors. However, several limitations hampered a full assessment. From the 17 risk factors eight were excluded in the first step because the exposure was too low to quantify the health impacts in terms of summary measures of population health (Figure 1). The concentrations were below the limit of quantification or the reference value.

In the next step, the literature search, no suitable epidemiological studies to deduct ERFs were identified for four risk factors. For the remaining five risk factors the input data was gathered in step 3 and for these five risks factors the burden of disease was quantified in step 4.

### 3.1 First step: exposure assessment

In the first step, the exposures of the GerES V participants towards the selected risk factors were analyzed. For some risk factors, this step required some degree of specification. For example, benzene was measured in indoor air and also by the use of biomarkers in urine, such as N-acetyl-S-phenyl-L-cysteine (Table 2). Because most ERFs from epidemiological studies considered exposure in ambient air, we selected ambient air exposure for our analyses. Another example that required further specification were phthalates: in the GerES V study, the urine samples were analyzed for 21 metabolites of 11 different phthalates (27). We finally focused on the metabolites with the highest concentrations in urine, namely Mono-iso-butyl phthalate (MiBP) and Mono-ethyl phthalate (MEP).

**Table 2:** GerES V exposure data.

Risk factors	Specification	Matrix	*n*	Limit of quantifycation	Median exposure	Comments	Citation	Next step
Arsenic		Urine	2,250	0.6 μg/L	9.06 μg/L		(53)	+
Benzene	N-acetyl-S-phenyl-L-cysteine	Urine	2,260	0.02 μg/L	0.097 μg/L	Limited epidemiological studies	(53, 79)	–
		Indoor air	615	1 μg/m^3^	1.10 μg/m^3^		(80)	+
Bisphenol A (BPA)		Urine	515	0.50 μg/L	1.905 μg/L		(53, 81)	+
Cadmium		Urine	2,250	0.05 μg/L	0.072 μg/L		(53, 82)	+
		Blood	720	0.12 μg/L	Below LoQ	limited epidemiological studies	(53, 82)	–
Chromium VI	Creatinine-adjusted	Urine	2,250	μg/L μg/L	0.34 μg/L	No differentiation between chromium species in urine data	(82)	–
Formaldehyde		Indoor air	639	0.7 μg/m^3^	24.9 μg/m^3^	Only 0.3% (n = 2) of sample with concentrations above the reference value of 100 μg/m^3^	(83)	–
Lead		Blood	720	2.1 μg/L	9.47 μg/L	No suitable health data available	(53, 82)	–
Naphthalene		Indoor air	615	0.6 μg/m^3^	Below LoQ	None of the samples above the reference value of 0.01 mg/m^3^	(84, 85)	–
Particulate matter	PM_2.5_	Indoor air Outdoor air	77 75	N.A.	10 μ/m^3^ 14 μg/m^3^	Small sample size		+*
Perfluorinated organic compounds (PFC)	Perfluorooctanoic acid (PFOA)	Blood plasma	1,109	0.50 μg/L	1.124 μg/L	Not enough project resources for this big group of substances	(53, 86)	–
Phthalates	MiBP	Urine	2,256	1.0 μg/L	26.06 μg/L		(27, 53)	+
	MEP	Urine	2,256	1.0 μg/L	25.8 μg/L		(27, 53)	+
Polychlorinated biphenyls (PCB) as a summation		Blood plasma	1,135	0.10 μg/L	0.303 μg/L	Only 0,1% above HBM-I (3.5 μg/L)	(53, 87)	–
Secondhand smoke	“More than one smoker in household”	Questionnaire	2,260	—	31.8%			+
	“Smoking mother“	Questionnaire	2,260	—	16.7%			–
	cotinine	Urine	2,260	0.1 μg/L	0.349 μg/L		(53, 88)	–
Terpenes	*α*-Pinen	Indoor air	615	0.6 μg/m^3^	6.4 μg/m^3^	Not enough project resources for this big group of substances	(84)	–
	*β*-Pinen		176	1.0 μg/m^3^	1.8 μg/m^3^			
	*δ*-3-Caren		355	0.75 μg/m^3^	2.3 μg/m^3^			
	Limonen		615	0.68 μg/m^3^	12 μg/m^3^			
	Longifolen		176	5.0 μg/m^3^	Below LoQ			
Traffic noise		At the window	2,126		49.64 L_eq_ [dB(A)] (15 min)		(44)	+*
TVOC		Indoor air	579		270 μg/m^3^	No suitable epidemiological studies	(84)	–
Ultrafine particles		Indoor air	2,172	—	5,470 cm^3^		(54)	+

LoQ, limit of quantification; N.A., not applicable; + considered further in step 2; – not considered further in step 2; *considered further, but exposure data from GerES was not suitable for exposure assessment.

Table 2 outlines the range of measurements in GerES V along with how we managed the data with the aim of using it for quantifying the EBD. It presents the matrices in which the risk factors were assessed in GerES V, including the number of analyzed samples and additional statistical measures to characterize the exposure. The table also includes a rationale for inclusion or exclusion of the risk factor in the further EBD process of our project and the final decision about inclusion. Further detailed information about the analytical methods in GerES V can be found in the citations for each risk factor displayed in the column citation.

Nine risk factors were considered for further analyses. The main reason for exclusion was too low exposure measured in GerES V and missing suitable epidemiological data.

### 3.2 Second step: literature search and selection of risk estimates

In the second step a systematic literature search was performed to identify ERFs for

- Arsenic
- Benzene
- Bisphenol A
- Cadmium
- Phthalates
- Secondhand smoke (based on systematic reviews)
- Ultrafine particles (PM_0.1_)

For PM_2.5_ and traffic noise existing reviews of the GBD-2019-study and WHO were used, respectively.

The risk factor specific search is explained in the following together with its results. The search for secondhand smoke differed, being an umbrella review not considering single studies.

#### 3.2.1 Arsenic

For arsenic the health outcomes skin, bladder and lung cancer were considered. In contrast to the search procedures for other risk factors, here, 100 titles were repeatedly reviewed by two reviewers. Once 90% agreement (Cohen’s kappa) had been reached regarding inclusion or exclusion of titles, the remaining articles were evaluated by only one person. This was issued due to time restriction in the project.

A total of 3,049 publications were identified. Of these, 1,834 were potentially suitable for inclusion after duplicates were removed. After reviewing the titles and abstracts, 321 publications were finally selected for full-text review. Eight additional publications were identified by a hand search. Of the total 329 full texts, 14 were finally classified as relevant according to the research question.

However, the effect measures derived in the epidemiological studies for the association of the respective health outcomes with arsenic were, in most cases, not statistically significant, and in some cases, even inconsistent. Furthermore, all studies focused exclusively on adults, due to the often long latency periods in cancer development. Therefore, no risk estimate for arsenic was available for the further analyses in the project.

#### 3.2.2 Benzene

For benzene a systematic search for the health outcomes leukemia, hematotoxicity and immunotoxicity was performed. However, studies investigating hematotoxicity and immunotoxicity were excluded from the subsequent burden of disease calculation because both effects represent preclinical changes in bodily systems, they are not quantifiable in terms of DALYs.

For the health outcome leukemia, a total of 3,950 articles were identified, of which 1,765 were potentially suitable for inclusion after excluding duplicates. The result of title screening yielded 601 relevant publications. Following abstract screening, 181 articles were found to be relevant, out of which 81 were identified as reviews. After full-text screening, 26 articles were included.

Most of the identified studies on leukemia were relatively old (published prior to 2000) and predominantly focused on occupational exposure, which, in most cases, was not directly measured but estimated by experts based on the work-related activities of the workers. The exposure to benzene in the work-related studies is considerably higher than the concentrations in indoor air as measured in GerES V. Consequently, the WHO indoor guideline values were used: a unit risk for benzene exposure with an additional lifetime risk for leukemia of 6 × 10^−6^ per 1 μg/m^3^. The corresponding lifetime risk is 1/10,000, 1/100,000, and 1/1,000,000 at benzene indoor air concentrations of 17, 1.7, and 0.17 μg/m^3^, respectively (28).

#### 3.2.3 Bisphenol A

Prior to the search, 13 health outcomes associated with bisphenol A exposure were selected and they formed the basis for the search strings. The search yielded 25,371 hits in total. However, initial checks revealed that the hits included too many studies that were not relevant, such as animal or toxicological studies. Therefore, the search was adjusted and bisphenol A was combined with the search term “epidemiological study.”

The search yielded three studies for obesity, two for asthma, two for coughing, one for depression and one for attention-deficit hyperactivity disorder (ADHD). Based on our *a priori* defined criteria to only quantify the burden of disease if at least three studies are available for one health outcome, only obesity was selected for further analyses. However, the studies used different exposure assessment methods for obesity (continues and categorical scale), therefore a pooling was not possible. The most recent study was thus selected for the next step of the analyses with an odds ratio ranging from 1.73 (1.16–2.58) to 2.05 (1.38–3.04) depending on the quartiles of the bisphenol A exposure (29).

#### 3.2.4 Cadmium

To identify any specific health effects that may be relevant to children and adolescents and to identify the relevant key words for the search string, a review of the available literature was conducted (30–33). The following health outcomes were identified: renal impairment, effects on cognitive abilities as well as behavioral problems. These include neurodevelopmental disorders, ADHD and autism spectrum disorder.

We identified 1,089 publications from which 757 were potentially suitable for inclusion after duplicates were removed. After title and abstract screening, 64 publications remained for full text review. Five additional publications were identified via hand search. Of the total 69 full texts, five were finally classified as relevant according to the research question. However, none of these studies reported significant ERFs. Thus, no risk estimate was selected for cadmium.

#### 3.2.5 Particulate matter

A literature search for PM was not conducted due to two main considerations. First, the extensive body of available studies and existing reviews on the subject did not favor any additional review. Second, our intention was to closely adhere to the methodology used in the GBD-2019-study to ensure methodological consistency and facilitate comparison of the results. Therefore, we also focused on the fraction PM_2.5_ and used the integrated ERFs derived from the GBD-2019-study for lower respiratory tract infection (LRTI), because this was the only health outcome relevant for children.

#### 3.2.6 Phthalates

The search for phthalates was conducted by a contracted external research team. A total of 22 relevant publications were identified, the majority of which were cohort studies. In terms of health outcomes, the studies considered growth/metabolism, sexual development/reproductive health, atopic diseases and neurocognitive development. The identified studies were characterized by a high degree of heterogeneity, in particular concerning the age groups, the phthalate exposures assessment and the health outcomes and data analysis methods. Due to the considerable heterogeneity, no synthesis of the results was performed and no risk estimate was selected for phthalates. The details of the literature search can be found in the UKAGEP-report (19).

#### 3.2.7 Secondhand smoke

Due to the high number of individual studies concerning secondhand smoke effects on health, our search efforts concentrated on systematic reviews. Eleven health outcomes were considered in the systematic search: asthma, bronchitis, heart disease, cancer, leukemia, lung cancer, neurodermatitis, otitis media, pneumonia, stroke and sudden infant death syndrome. The initial search resulted in 4,810 hits. After removing duplicates and screening the titles, 1,100 were considered relevant. The publication date was restricted and only articles published after 2012 were included. After this restriction 51 relevant articles were identified. Of these 34 were rated of good quality and therefore considered further in the process. Two were excluded *post-hoc* because no meta-analysis was conducted.

The meta-analyses were highly heterogeneous. Among other aspects, they referred to different target groups, different definitions or categories of secondhand smoke exposure or health outcomes and often summarized results of different effect measures from different study designs. Therefore, we decided not to combine the meta-analyses into a single risk estimate but to select the meta-analysis with the highest quality. Furthermore, the studies with the largest number of individual studies included and studies conducted in Europe were therefore favored. The finally used effect measure and their 95% confidence interval are 1.32 (1.23–1.42) for asthma (34), 1.06 (1 0.01–1.11) for atopic dermatitis (35, 36), 1.09 (1.04–1.14) for allergic rhinitis (hay fever) (35, 36), 1.32 (1.20–1.45) for otitis media (37), 1.43 (1.28–1.59) for LRTI (38), 1.97 (1.77–2.19) for sudden infant death syndrome (39) and 1.59 (1.17–2.15) for invasive meningococcal disease (40).

#### 3.2.8 Traffic noise

For traffic noise we did not conduct a review but relied on the noise guidelines published by the WHO, which are based on comprehensive systematic literature searches (41). Traffic noise is categorized in road traffic, rail and aircraft noise. According to the guidelines, children are a particularly vulnerable group. The considered health effect is “reading skills and oral comprehension in children” due to aircraft noise. The recommendations of Kamp et al. (42) were followed and the ERF from the cross-sectional RANCH-study was used. According to this, an increase of 10 dB(A) in aircraft noise at school is associated with a delay in learning to read between one and 4 months. The WHO considers delays in learning to read of one month or more as a significant health risk (41).

For the other two noise types (road and rail traffic), however, the evidence was rated insufficient. Other health outcomes for children have been studied (emotional and behavioral disorders, hyperactivity, hypertension, memory impairment), but the evidence for the association with traffic noise was rated as very low.

#### 3.2.9 Ultrafine particles

The search for ultrafine particles was conducted by a contracted external research team and the results are published in a separate article (43). Health outcomes considered in the review were mortality, morbidity (e.g., cardiovascular and respiratory disease), hospitalizations/emergency room visits and prehospital outcomes. The majority of studies identified preclinical effects, amounting to approximately 60% of the effects. The systematic review yielded no health outcomes for which the association with ultrafine particles was supported by sufficient evidence, and thus no risk estimate was selected for ultrafine particles.

#### 3.2.10 Summary

For arsenic, cadmium, ultrafine particles and phthalates no suitable effect measures related to the child or adolescent population was identified in the systematic search. Thus, these risk factors were not considered in the burden of disease quantification.

### 3.3 Third step: input data

In the third step, the input data were collected. Aside from traffic noise and PM_2.5_, the exposure data were based on GerES V survey data. For both risk factors the measurement period in GerES V was too short for a representative assessment of long-term exposure (noise 15 min and PM_2.5_ 7 days).

For noise, the EU Environmental Noise Directive (2002/49/EC) offers comprehensive data. Noise maps are available for all major roads, major railways, and major airports as well as agglomerations with more than 100,000 inhabitants in Germany and the reference year 2016. Noise is assessed in the indicator L_den_ for the day-evening-night noise and L_night_ for the night-time noise. The Common Noise Assessment Methods in the EU (CNOSSOS-EU) is used for the calculation procedures for traffic noise and noise from industry. More details can be found in Tobollik et al. (44).

The exposure assessment for PM_2.5_ is described in Tobollik et al. (45) and Kienzler et al. (46) in detail. In summary, PM_2.5_ concentrations were derived from a model of nationwide annual mean PM_10_ rural and urban background concentrations, using a fixed conversion factor of 0.7, to arrive at nationwide annual mean PM_2.5_ concentrations with a spatial resolution of approximately 2 × 2 km^2^ for the year 2016. These data were combined with spatial population density information from the 2011 Census to estimate population-weighted exposure.

Population data as well as data on life expectancy were obtained from the German Federal Statistical Office (47, 48). Mortality data were retrieved from the cause-of-death statistics for selected International Statistical Classification of Diseases (ICD)-10-codes published by the Federal Statistical Office in the German Federal Health Monitoring (49). Cause-of-death data were used as reported without correction of garbage-codes. As there is no comparable register for prevalent or incident cases in Germany, the data for obesity, atopic dermatitis, allergic rhinitis (hay fever), asthma, otitis media and obstructive/spastic bronchitis were gathered from KiGGS. And data for the number of deaths due to meningococcal infection was gathered from German health reporting (49).

Disability weights were obtained from the GBD-2019-study (50). For each health outcome we calculated the age and sex-specific disability weights by dividing the annual years lived with disability by the prevalence data. Using this approach allowed to use co-morbidity and severity-adjusted disability weights for Germany.

### 3.4 Forth step: environmental burden of disease quantification

In the final step the environmental burden of disease was quantified. This was possible for five risk factors: benzene, bisphenol A, PM_2.5_, secondhand smoke and traffic noise. In total, 11 risk-health outcome pairs could be considered (Table 3). However, different quantification methods were applied or not the full range of classic EBD indicators was calculated, such as PAF, YLLs and YLDs. Furthermore, the age ranges differ due to the availability of the ERFs and the specific age groups to which each ERF is applicable. The risk factor specific results are described in the following sections.

**Table 3:** Estimated EBD indicators for risk factors and associated health outcomes.

Risk factor	Health outcome	Age group (years)	EBD indicators quantified			
			PAF	YLD	YLL	Others
Benzene	Leukemia	3–17				Unit risk (morbidity)
Bisphenol A	Obesity	7–17	x			Attributable cases (morbidity)
PM_2.5_	Lower respiratory tract infections	3–17	x	x		
Secondhand smoke	Allergic rhinitis (hey fever)	3–17	x	x		
	Asthma	3–17	x	x		
	Atopic dermatitis	3–17	x	x		
	Invasive meningococcal disease	3–17	x			Attributable cases (morbidity)
	Lower respiratory tract infections	<2	x	x		
	Otitis media	3–17	x	x		
	Sudden infant death syndrome	<1	x		x	
Traffic noise (aircraft)	Reading skills and oral comprehension in children	7–17	x			Attributable cases (morbidity)

PAF, population attributable fraction; YLD, years lived with disability; YLL, years of life lost due to mortality; x: were quantified.

#### 3.4.1 Benzene

The lifetime risk of leukemia is estimated to be approximately slightly above 1 person per 100,000, based on the WHO unit risk of 6 × 10^−6^ per 1 μg/m^3^ and the exposure of 1.81 μg/m^3^ benzene in indoor air, as determined in GerES V. For the population of 3 to 17-year-olds, the calculated number of statistical cases of leukemia is 112, with a lifetime prevalence of 70 years and continuous exposure of 1.81 μg/m^3^. The conversion into units of YLL, YLD or DALY was not possible.

#### 3.4.2 Bisphenol A

The calculation was conducted for all individuals between the ages of 7 and 17 years who exhibited bisphenol A concentrations in their first morning urine exceeding 1.3 μg/L. This concentration aligned with the reference quartile used in the study by Eng et al. (29).

With regard to the health outcome obesity, no disability weight was available in the GBD-2019-study, which limited our assessment. Furthermore, we assumed that no children are dying because of obesity, therefore no YLLs were quantified. Consequently, only the prevalent obesity cases attributable to bisphenol A were estimated.

A population attributable fraction of approximately 28% (95% CI 9.3–40.8) was estimated. This indicated that around 28% of obesity cases in children and adolescents aged 7–17 years could be attributed to bisphenol A exposure, with no considerable differences between boys and girls. In total numbers, approximately 171,130 (95% CI 56,247-247,809) children and adolescents in Germany are affected by bisphenol A related obesity. To our knowledge, there are no other burden of disease estimates for bisphenol A exposure in Germany.

#### 3.4.3 Particulate matter

For PM we decided to only quantify the burden due to PM_2.5_ exposure, as evidence in this field has increased in recent years and more epidemiological studies are now available for this fraction. Furthermore, we followed the approach of the GBD-2019-study. In the GBD-2019-study, only LRTI is quantified as a health outcome for children. We used the ERF from the GBD-2019-study.

The calculation was carried out for all persons aged 3 to 17 years who were exposed to concentrations above 4.2 μg/m^3^ PM_2.5_ as an annual average at their place of residence in 2016. The PAF was calculated for the age groups 0–10 and 10–20 years, as the PM_2.5_ data was available in these age groups. The burden of disease was calculated according to the data available in GerES V for each age group from 3 to 17 years. The PAF was estimated at 4.7 (95% CI 2.6–7.3), indicating that 4.7% of LRTI cases are attributable to PM_2.5_ exposure. In total, 1,342 (95% CI 774–2,088) DALYs were lost in 2016 due to morbidity from LRTI.

In 2016, no children between the ages of 3 and 17 died from LRTI. For this reason, the YLLs were not calculated. The DALYs therefore correspond to the YLDs for this health outcome.

Unfortunately, the GBD study results were not available in exactly suitable age-groups for comparisons. Thus, our comparator was the age-group 1–19 years. Even though this age-group is wider, the YLD attributable to PM_2.5_-exposure in the GBD 2019 study are far lower with 25 YLD. In contrast to our assessment, mortality effects were considered in the GBD study which in addition led to 191 YLL, resulting in an overall attributable burden of 216 DALY. Only comparing the YLD, we consider the different sources for the prevalence data as the major reason for the observed differences. In our study we used self-reported data from the GerES V study which resulted in 452,426 prevalent cases in the age-group 3 to 17 years. In the GBD 2019 study 6,649 prevalent cases for the age-group 1–19 years were estimated based on a complex model which combined different data sources. The discrepancies can be explained by different case definition for a LRTI case in the relevant data sets.

#### 3.4.4 Secondhand smoke

For secondhand smoke risk estimates for seven health outcomes were selected, including atopic dermatitis, allergic rhinitis (hay fever), asthma, otitis media, LRTI, sudden infant death syndrome and invasive meningococcal disease. A detailed explanation of the methodology and EBD results will be published in a separate article.

#### 3.4.5 Traffic noise (aircraft)

For air traffic noise, only the PAF was calculated for the outcome “reading skills and oral comprehension in children,” as no reliable prevalence data on this effect is available for the population of children in Germany.

The classic EBD indicators were not calculated because reading literacy and listening comprehension are no clinical health outcomes where disability weights indicate the severity of the health impact. Thus, also no prevalence data is available for these outcomes. Instead, only the proportion of children who have impairments in their reading skills and listening comprehension that can be attributed to air traffic noise was determined. As there is a lack of detailed data on the distribution of exposure levels to aircraft noise among children in Germany, we assumed as an approximation that children are exposed to the same extent as the overall population. The lower quantification limit used was 50 dB(A), as proposed by Kamp et al. (42).

2.4% of children were exposed to noise levels above 50 dB(A), resulting in a one-month delayed acquisition of reading skills in 0.67% (95% CI 0.11–0.73) of children living in Germany. In absolute terms, these are 4,788 (95% CI 786–5,217) of the 714,614 seven-year-old children in 2016.

Burden of disease studies on traffic noise mostly focus on adult populations (51, 52). Due to our knowledge, there are no studies focusing on children and traffic noise in Germany.

## 4 Discussion

This discussion focuses primarily on the methodological challenges we encountered during the steps of the EBD estimation. Giving the limited number of comparable studies, particularly those focusing on children in Germany or Europe, comparison with other EBD studies is limited. We therefore include suggestions that may support and inform future EBD assessments.

### 4.1 Limitations in the four quantification steps

Our aim was to estimate the burden of disease for 17 environmental risk factors using the same methodology to achieve comparability of results. However, during the project, several obstacles hampered the achievement of our objective. In the end, the burden of disease calculation processes varied considerably for each risk factor. The reasons for this discrepancy are manifold and can be traced back to various components of the calculation processes, which will be explained in the following sections.

**Figure 2:**
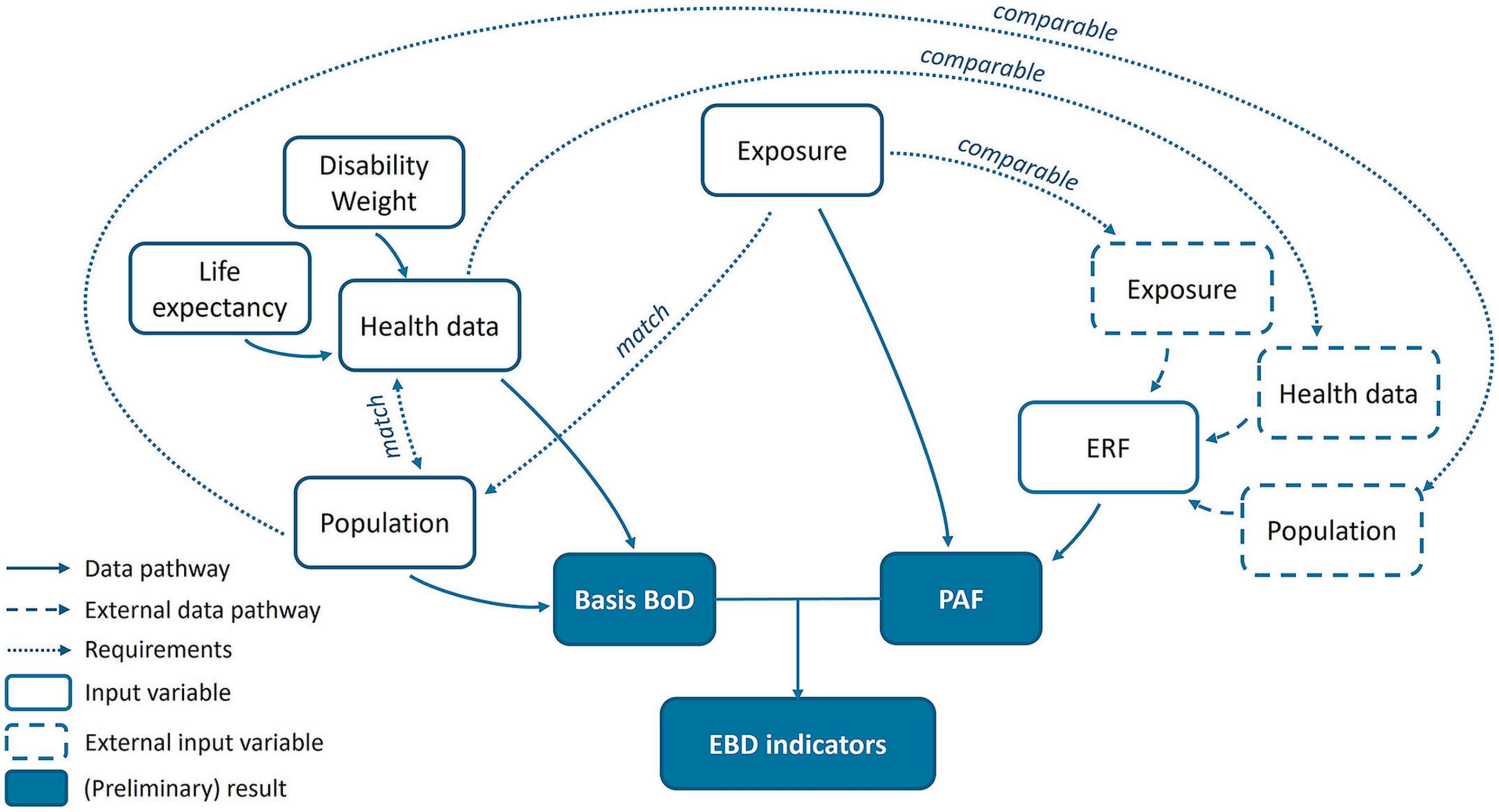
Dependencies in the data framework of EBD studies, here using the example of the UKAGEP project.

Figure 2 schematically illustrates the necessary input data and their subsequent processing for calculating disease burden indicators (DALYs, YLDs, YLLs, PAF). In EBD studies, a variety of input data are required, and these must be compatible with each other, resulting in dependencies in data selection (4). For example, the exposure data must match with the population data, meaning the exposure data must be available for a specific population (group), in our case, children. If exposure data were not available for this population group, the disease burden could not be calculated. Similarly, the health outcomes for which the ERF is available must be comparable with the health data used (26).

We aimed to ensure that our estimates were as comparable as possible to those of the GBD study, while focusing on using Germany-specific data. Given the GBD study as a remarkable and unique assessment its global scope is to measure and analyze the worldwide impact of diseases, injuries, and risk factors. In doing so, all results of the GBD study are based on models using a variety of assumptions and do not necessarily correspond to country-specific information or raw data collected in these countries. Therefore, there is still a need for country-specific studies such as presented by our assessment, because they incorporate local data and account for risk factors that, although not included in the global analysis, may play a crucial role at national level. In our study, we used specific health and human biomonitoring data, which are not available for all countries globally, to test whether these can be effectively incorporated into EBD quantifications. Therefore, national EBD studies may provide more meaningful insights for that specific country than the results of the GBD study.

#### 4.1.1 Exposure

The primary source of exposure data was the GerES V study (53). However, in the end GerES V data was only suitable for the exposure assessment regarding arsenic, benzene, bisphenol A, cadmium, phthalates and secondhand smoke. For PM_2.5_ and traffic noise different sources of exposure data were used and for ultrafine particles no suitable exposure assessment was conducted with the available GerES V data (44, 45).

For population representative PM_2.5_ levels and traffic noise data, we relied on additional data sources. In the GerES V study, PM_2.5_ was measured indoors and outdoors in 74 households over a seven-day period. The brief measurement period does not provide a reliable representation of exposure, but is rather a snapshot that is prone to multiple confounding factors. Furthermore, the number of households is insufficient to derive an average exposure estimate representative for the German population. Therefore, we used German wide modeled exposure data (45, 46).

The noise assessment in GerES V was a short-term measurement of 15 min, which is not representative of the average exposure of individuals living in the household over a longer period of time. To quantify the health impacts in terms of summary measures of population health, it is necessary to have access to long-term environmental exposure data. Exceptions to this rule are very high short-term exposures. Such high concentrations were not identified in GerES V.

In GerES V, ultrafine patricles were measured, but the measurement duration was relatively short (1 h), which means the measured data cannot be considered representative for long-term exposure (54). They provide indicative information and only, on average, give a meaningful indication of exposure to ultrafine particles indoors. Such measurements are not suitable for individual-level assessment nor for population-based long-term exposure assessment.

For most risk factors, the exposure levels assessed in GerES V were below the threshold from which we would assume an increased risk and burden of disease (53). The thresholds are mostly the limit of quantification or the reference values. This holds for formaldehyde, naphthalene and polychlorinated biphenyls in our assessment. This does not imply that their exposure is uncritical or that no negative health impacts are possible. It is simply that, based on the current state of knowledge, no EBD can be quantified due to the low level of exposure. Furthermore, in addition to indoor air, there are other sources of exposure to formaldehyde, naphthalene, and benzene that can increase children’s health risk, including toys, outdoor air, and dust, meaning we likely underestimate both their exposure and, consequently, their burden of disease.

The exposure data are assessed specifically for the German context. A generalization is therefore limited. Projects such as the European Human Biomonitoring Initiative (HBM4EU) aim at assessing the body burden of a huge range of risk factors for European countries. This is an excellent data basis for a European environmental burden of disease study, as has been and is still being carried out within the framework of the European Environment Agency’s European Topic Centre on Human Health and the Environment (55, 56).

**Suggestions for improvement:**

- Begin by checking the exposure data: ensure it aligns with the target population, the matrix (such as human biomonitoring, indoor air, drinking water, etc.), the units of measurement, and the exposure duration. Then adjust your literature search to obtain specific ERFs.
- Optionally the other way around: start with the ERF and search for suitable exposure data.
- Perform validity checks including whether the exposure data matches with the exposure data in the epidemiological studies, such as exposure duration, confounder assessment and the range of exposure.
- Ideally, use ERFs derived from multipollutant assessments to better represent the complexity of human exposure.
- Ideally, stratify by subgroups, such as sex, age groups, socio-economic groups, to identify stronger affected, higher burdens or vulnerable groups.

#### 4.1.2 Exposure-response function

The ERF is a crucial model input for the EBD quantification. It is a decisive factor in determining the level of the disease burden (57). Therefore, the selection of the ERF should be done with caution. We performed systematic literature searches and additionally relied on systematic searches performed by the WHO or within the GBD-2019-study. However, the evidence for the ERF of the five risk factors varies, which hampers the comparison of their burden of disease. For instance, the evidence for PM_2.5_ is far more advanced compared to bisphenol A. While the unit risk for benzene and leukemia is widely accepted, additional health outcomes related to benzene would also be of interest. To put it in a nutshell, the lack of ERF data and the varying quality of the available evidence limit the comparability of these risk factors.

When conducting our own literature search, we set a minimum criterion of at least three studies to ensure robustness. However, in practice, this proved challenging because many studies used varying definitions [e.g., for bisphenol A: exposure was assessed dichotomously (58) or continuously (59)] and had differing areas of focus, making comparison or even pooling nearly impossible. Despite these challenges, we evaluated the quality of all identified studies using validated assessment tools.

In the literature searches, the identified ERFs were sometimes contradictory, not statistically significant [e.g., for cadmium (60, 61), or skewed by confounding factors (62)]. In addition, some associations on health effects have so far been derived predominantly from non-human studies (e.g., for bisphenol A or phthalates). It would be beneficial to have a greater number of high-quality epidemiological studies on health effects for humans to estimate the burden of disease more accurately (63).

The systematic literature reviews revealed that only a few suitable ERFs were available for our target group, children and adolescents. It can therefore be assumed that the burden of disease tends to be underestimated in general and also in our study. We chose not to apply ERFs derived for adults to our analyses for children, despite the awareness that this limits the scope of our assessment. Using adult ERFs could have provided more comprehensive EBD estimates, but also introduce uncertainties. Frequently, the exposures to risk factors investigated in the identified epidemiological studies, especially from the Asian region, were in part higher than the exposures measured in Germany. Consequently, the transferability of the identified ERFs to Germany is often only possible to a limited extent. Nevertheless, we assumed transferability of ERFs that were not primarily derived for Germany, as the biological patterns of the effects of exposure on health should not differ regionally. Therefore, we see a possible generalization of the applied ERF to other, probably European, contexts.

For phthalates and ultrafine particles, the literature search revealed a lack of epidemiological studies especially for children and adolescents. Thus, no ERFs were extracted. For arsenic and cadmium, epidemiological studies on cancer outcomes were available, but the results were not statistically significant [e.g., for arsenic (64–66)]. Therefore, the restricted availability of suitable ERFs significantly constrained our EBD assessment, especially for the risk factors TVOC (67) and ultrafine particles (43).

A further limitation is that some ERFs are only available for morbidity or mortality. However, the application for both components as an approximation is not suitable for all health outcomes (4). Therefore, we only used the respective ERF for which it was derived, morbidity or mortality. This approach differed from that of the GBD-2019-study, which uses the same ERFs for estimating the PAF for YLDs and YLLs, thus yielding a higher number of DALYs (68).

**Suggestions for improvement**:

- Indicating the level of evidence for the association between exposure and health outcome to ensure transparency.
- Evaluating the quality of studies using validated assessment tools.
- Deciding *a priori* for a minimum of available studies (preferably epidemiological studies) to be certain about the association.
- Check if other institution provide ERF to ensure comparability and to avoid double work.
- If doing an own literature search follow the Cochrane guidelines.

#### 4.1.3 Health outcomes

KiGGS 2 was the primary source of health outcome data (21). To combine health outcome data from KiGGS 2 and the ERFs, it is crucial to apply consistent definitions across both datasets. Divergent definitions of health outcomes can lead to a misclassification and thus skew the burden of disease results. In order to allow a correct merging of the different health data, the application of ICD codes in order to define health outcomes in epidemiological studies would be helpful. However, in our case the majority of the selected studies did not include ICD codes. Therefore, we used the ERFs for health outcomes, which were not specifically tailored to the selected health outcomes. For example, for secondhand smoke and PM_2.5_, the ERF for LRTI was used on obstructive/spastic bronchitis data (3).

Another challenge was that in many epidemiological studies identified in the literature search, health impairments (e.g., impaired lung or kidney function) are recorded, although these do not necessarily lead to clinically relevant diseases and thus no quantification in terms of summary measures of population was possible [e.g., for cadmium (69, 70)]. In addition, the studies considered used different data sources (e.g., cancer registries, population studies) with different methods to operationalize ascertainment for outcomes, e.g., biomarkers, physician diagnoses, or self-reported information as was the case with pooled individual studies in meta-analyses on secondhand smoke, for example: Murray et al. (40) and Saulyte et al. (36). This also limits the comparability of results and complicates the pooled evaluation of studies in meta-analyses.

It should also be noted that obtaining data on morbidity in Germany is much more difficult compared to mortality data because, unlike the death statistics, there are no registries for this kind of data—except for cancers.

The morbidity and mortality data are specific for Germany. A generalization to other countries should not be done, because the data can differ a lot.

**Suggestions for improvement:**

- Using ICD coding to combine morbidity or mortality data and ERFs.
- Using official country data, if available (e.g., disease or death registries).
- Considering redistribution of garbage codes in the death statistics, as done by the GBD study if comparison with GBD is favored.
- Emphasizing the need of prevalence data for research purpose on national level.

### 4.2 Discussion—risk factor specific

Of the 17 environmental risk factors initially considered, it was ultimately only possible to calculate the disease burden for children and adolescents in Germany for five factors: benzene, bisphenol A, PM_2.5_, secondhand smoke and traffic noise by aircrafts. The risk factor specific limitations are explained in the following.

#### 4.2.1 Benzene

Benzene is a carcinogenic chemical that has been subject of extensive research. EBD calculations on benzene were carried out by Hänninen et al. (71) and Hornberg et al. (51) for Germany, however, children were not the focus of these studies. The reason given in the latter publication is that children are not considered to have a higher susceptibility (51). The literature search yielded only studies that conducted workplace exposure assessments for benzene. The same applies to the most recent evaluation of the GBD-2019-study, which focuses exclusively on occupational exposure to benzene (72).

Consequently, the well-researched unit risk method was used to calculate the burden of disease. Although the unit risk for benzene is an established risk estimator, it is already quite old. Nevertheless, it is assumed that it is still up to date and valid, as the WHO has not revised this value and refers to it in current publications (73). Nevertheless, the project team considers a confirmation of the value to be desirable. Furthermore, the applied method and the results for benzene are not directly comparable with the other burden of disease methods and estimates because it refers to a lifelong (70 years) exposure. A differentiation between YLL and YLD was not possible, and only attributable cases based on the assumption that the children and adolescents will be exposed to the same exposure for their lifetime were quantified.

#### 4.2.2 Bisphenol A

The systematic review for bisphenol A mostly identified cross-sectional studies. This study design does not per se allow any conclusions about a causal relationship between bisphenol A and specific health outcomes such as obesity. This also applies to the study from which the ERF for burden of disease quantification was selected. A validation of the relationship based on longitudinal studies is necessary to improve the evidence base of the risk estimates (74). Furthermore, it was not possible to pool the available effects measures from the studies on bisphenol A and obesity, due to differing definitions of exposure employed in the studies. Thus, the quantification was based only on a single effect measure.

Another limitation of the bisphenol A analysis is the exposure assessment in the selected study, which was a single sampling. Bisphenol A measurements are highly variable in terms of differences in assessment time (within a day and on different days) (75). The single sample therefore carries a considerable degree of uncertainty when assessing or classifying the individual bisphenol A exposure of the study participants (75–77). Overall, the results for bisphenol A should therefore be interpreted with caution.

The missing disability weight for obesity further hampered the quantification of the burden of disease of bisphenol A in terms of YLDs.

#### 4.2.3 Particulate matter

The exposure assessment for PM_2.5_ refers to annual averages ambient outdoor air concentrations, which is a reliable indicator for long-term exposure. However, most health effects related to long-term exposure of PM_2.5_ are chronic diseases, which occur after long exposure periods of many years. Effects relevant for children and adolescents are rather short-term. Nevertheless, we used the annual PM_2.5_ exposure as an approximation to estimate the YLDs due to LRTI.

#### 4.2.4 Secondhand smoke

Secondhand smoke is an extensively researched risk factor. Therefore, the umbrella review focused on already available systematic reviews and meta-analyses and not on single studies. Secondhand smoke was the risk factor with the most associated health outcomes in the underlying assessment. These health outcomes included atopic dermatitis, allergic rhinitis (hay fever), asthma, otitis media, LRTI, invasive meningococcal disease and sudden infant death syndrome.

In GerES V, several indicators for secondhand smoke exposure were assessed from different questions in the questionnaires in addition to human biomonitoring (HBM) data. The most accurate approximation of secondhand smoke exposure are HBM data. However, we derived exposure from the questionnaire, reporting whether at least one person was smoking in the household. This exposure definition corresponded to the majority of the systematic reviews identified in the umbrella review. However, this definition does not allow for differentiation between a person smoking inside or outside of the home or how much the person is smoking.

#### 4.2.5 Traffic noise (aircraft)

Previously, the burden of disease caused by traffic (road, air and railway) noise was quantified specifically for Germany in two studies (51, 78). However, these studies focused on high annoyance and sleep disturbance and did not consider children. This is because children feel less annoyed by noise and fewer studies have been carried out on sleep disorders caused by noise in children. Furthermore, it should be noted that extra-aural exposure to noise requires a certain exposure time before health effects become apparent. In comparison, the health outcomes of children’s reading skills and listening comprehension, in which were the focus of our study, are less well researched. One limitation of our study is that we combine modeled aircraft noise data for the general population with an ERF specific to aircraft noise at schools.

### 4.3 Policy implications

Our study highlights the critical importance of high-quality research data, including prevalence data, death statistics, demographic data, exposure data and other epidemiological parameters for accurately estimating the burden of disease in terms of summary measures of population health. A wide range of input data is necessary to quantify the environmental burden of disease. When data are lacking, the precision of burden of disease estimates is limited, which restricts one of the key advantages of these estimates—comparing the impacts of different risk factors to prioritize public health topics.

Policy makers need to know the greatest (environmental) threats for population health. The burden of disease methods can, in some circumstances, provide an answer to this question. To do this, it is important to consider all potential risk factors and to ensure that the input data for each factor are comparable and available in good quality. We demonstrated that for nearly all of the 17 risk factors, additional input data would improve the accuracy of the estimates.

Similar to the GBD-2023-study, our research identified that the highest environmental burden was associated with PM_2.5_ exposure (18). However, this finding is partly due to the fact that air pollution is one of the most extensively researched environmental risk factor. Numerous burden of disease estimates for PM_2.5_ highlight its significant impact, but this does not necessarily imply that it carries the highest overall burden, as the extensive research on air pollution may skew its relative importance compared to other, less studied environmental risk factors. Air pollution is also a key political concern, as reflected in the revised, stricter European Air Quality Directive. In the development of this legislation burden of disease estimates were used to demonstrate the potential health benefits of enforcing stricter air quality limit values, serving as an evaluation of policy effectiveness. We also want to emphasize the importance of not limiting the focus to well-researched and regulated environmental risk factors, such as PM_2.5_ and noise. It is crucial to also address emerging risk factors, such as perfluorinated organic compounds and to collect data on less studied pollutants to gain a more comprehensive understanding.

Additionally, our finding can support public health education by underscoring the importance of environmental protection and its impact on human health, fostering greater public health awareness. However, these conclusions are constrained by the limitations of the study. Nevertheless, the insights can be valuable to public health officials, environmental advocacy groups (such as NGOs), and healthcare professionals (including doctors, nurses, and other practitioners), who can use these findings to better inform the public and healthcare practices about the impact of environmental factors on health.

Our results can also help to further foster measures on the road to pollutant free Europe as indicated in the Zero Pollution Ambition Strategy, where the chemical loads in the environment would not harm human health.

## 5 Conclusions and suggestions for future research

A key strength of this study is that it is one of the few studies to date that has examined the burden of disease caused by environmental factors among children and adolescents in Germany. Additionally, the estimates are based on the standardised, well-established EBD method, and the exposure and health data are based on representative studies. However, the EBD method requires several input data that are not readily available for the EBD estimation for children in Germany. This makes a comprehensive overview and to compare or evaluate the results of their EBD challenging. Our aim was to use a standardized method for the 17 selected risk factors, which was not possible due to data limitations. In the end, the burden of disease calculations and the according results varied considerably for each risk factor. For many risk factors, the exposure levels assessed in GerES V were below the limit of detection or the reference value from which we would assume an increased health risk and thus burden of disease. However, this does not imply that the exposure is uncritical or that no negative health impacts can occur after a life-long exposure. It is simply that, based on the current state of knowledge, no EBD can be quantified due to the low level of exposure.

While individual datasets, such as the comprehensive GerES V study, were accessible, integrating them with other secondary data posed challenges due to the differing definitions of health outcomes or levels of exposure data. In Germany, there is a pressing need for more comprehensive health and exposure data collection efforts. This could involve the implementation of initiatives such as health surveys to gather morbidity data or the expansion of access to claims data for research purposes. Ideally, large-scale representative population studies should encompass both health and environmental dimensions, facilitating the establishment of connections between exposure and health effects at an individual level. The availability of more high-quality data on exposure, health, and their interrelationship, including ERFs, would significantly enhance the comprehensive understanding of the burden of disease in the German population.

**Suggestions for future research:**

- Enhancing the collection of prevalence data defined by ICD-codes on a regular basis, stratified by different age groups, sex and socio-economic status and grant access to the data for relevant stakeholder (e.g., scientists).
- Fostering the further improvement of the death statistics to minimize wrong or incomplete coding.
- Conducting longitudinal epidemiological studies to assess ERF, also stratified by different age groups, sex and if possible socio-economic status, especially for emerging and less-regulated pollutants.
- Fostering research on combined and cumulative effects of pollutants.

## Data Availability

The raw data supporting the conclusions of this article will be made available by the authors, without undue reservation.
