# Supplementary material for: Estimating the environmental burden of disease in children and adolescents in Germany: limitations and possible solutions
Source: Front Public Health. 2026 Apr 10;14:1796283. doi: 10.3389/fpubh.2026.1796283 (PMC13106403; doi:10.3389/fpubh.2026.1796283)
Supplement: Supplementary file 1 [file Supplementary_file_1.docx]

Supplementary Material

# General aspects on the literature search

The preparatory work for the literature search involved identifying German- and English-language search terms and combinations of search terms for the relevant risk factors. Based on information from existing publications by relevant institutions, including the World Health Organization (WHO) and the Agency for Toxic Substances and Disease Registry (ATSDR), search terms were developed for each risk factor under consideration. These terms included both the English terms for exposure and the corresponding health outcomes.

The search terms and search term combinations were developed in close consultation with experts in toxicology and human medicine at the German Environment Agency. The search strategy was subsequently reviewed on a sample basis by a librarian at the German Environment Agency.

## Inclusion criteria:

- Epidemiological studies with cohort, case-control, or cross-sectional designs
- Report of quantitative results in the form of effect measures (RR, HR, OR, SMR), or similar)
- Number of participants in studies N > 20
- English- and German-language articles; French-language articles only if the relevance could be clearly derived from the (translated) title

## Exclusion criteria:

- Studies on the effects of prenatal exposure of the mother or foetus
- Health effects corresponding to disease precursors
- Studies on occupational exposure
- Non-human studies, e.g., animal experimental studies (in vivo, in vitro)
- Studies focused solely on exposure, biomarkers, cells, or genetic influences
- Ecological studies if no effect measure is present or if no adjustment for confounding factors was made
- Systematic or narrative reviews or qualitative studies
- Grey literature, including conference contributions, opinion articles, letters to the editor, comments, errata (unless containing relevant information)
- Studies in all other languages

# Arsenic

The initial comprehensive list of possible health endpoints related to arsenic included: bladder cancer, skin cancer, lung cancer, liver cancer, kidney cancer, skin lesions, diabetes, neurotoxic effects, developmental disorders, IQ deficits, increased blood pressure, heart rate variability, ischemic disease, atherosclerosis, spleen effects, intravascular hemolysis, leukopenia, thrombocytopenia, respiratory diseases, asthma and renal dysfunction. However, to make the search more feasible for environmental burden of disease studies, the search terms were further refined by incorporating the terms epidemiological studies and exposure-effect relationships and by focusing on skin, bladder and lung cancer.

**Arsenic specific exclusion criteria**

- Arsenic exposure measured in hair or nails
- Studies on the therapeutic effects of arsenic in cancer treatment
- Studies on cancer development resulting from treatment with arsenic as a cancer therapy

In contrast to the search procedures for other risk factors, for arsenic 100 titles were repeatedly reviewed by two reviewers. Once 90 % agreement (Cohen's kappa) had been reached regarding inclusion or exclusion of titles, the remaining articles were evaluated by only one person. This was issued due to time restriction.

A total of 3,049 publications were identified. Of these, 1,834 were potentially suitable for inclusion after duplicates were removed. After reviewing the titles and abstracts, 321 publications were finally selected for full-text review. Eight additional publications were identified by a hand search. Of the total 329 full texts, 14 were finally classified as relevant according to the research question.

**Bladder cancer**

- Chen, Su (1)
- Chung, Huang (2)
- Huang, Huang (3)
- Huang, Pu (4)
- Melak, Ferreccio (5)
- Pu, Yang (6)
- Steinmaus, Bates (7)
- Wu, Chen (8)

**Lung cancer**

- Argos, Parvez (9)
- Chung, Huang (2)
- García-Esquinas, Pollán (10)
- Melak, Ferreccio (5)
- Steinmaus, Yuan (11)

**Skin cancer**

- Chen, Guo (12)
- Gilbert-Diamond, Li (13), Yu, Hsu (14)
- Leonardi, Vahter (15)

However, the effect measures derived in the epidemiological studies for the association of the respective health outcomes with arsenic were, in most cases, not statistically significant, and in some cases, even inconsistent. Furthermore, all studies focused exclusively on adults, due to the often long latency periods in cancer development. Therefore, no risk estimate for arsenic was selected for estimating the environmental burden of disease in children and adolescent.

# Benzene

In preparation for the search, three health effects associated with benzene exposure were identified by a toxicologist from the German Environment Agency as particularly relevant: leukemia, hematotoxic and immunotoxic effects. Consequently, these three health effects were defined as primary search terms. In the end, we chose to focus on leukemia, as the other two conditions are not directly quantifiable within the framework of the burden of disease concept.

**Example search term for leukemia:**

(benzene[Title/Abstract] AND leukemia[Title/Abstract]) OR (benzene[Title/Abstract] AND acute myelo* leukemia[Title/Abstract]) OR (benzene[Title/Abstract] AND AML[Title/Abstract])

(benzene[MeSH Terms] AND leukemia[Title/Abstract]) OR (benzene[MeSH Terms] AND acute myelo* leukemia[Title/Abstract]) OR (benzene[MeSH Terms] AND AML[Title/Abstract])

No special exclusion criteria were applied for benzene.

For the health outcome leukemia, a total of 3,950 articles were identified, of which 1,765 were potentially suitable for inclusion after excluding duplicates. The result of title screening yielded 601 relevant publications. Following abstract screening, 181 articles were found to be relevant, out of which 81 were identified as reviews. After full-text screening, 26 articles were included.

All studies were evaluated in terms of quality. For this, the evaluation tool of the National Heart, Lung, and Blood Institute was used. The quality assessment was carried out independently by two project staff members, with a high degree of agreement achieved. In case of discrepancies, the respective cases were discussed, and consensus was reached in all instances.

As a result, out of the 26 studies, 15 were rated as good, 9 as adequate, and two as poor. The studies rated as good are the following:

- Axelsson, Barregard (16)
- Collins, Anteau (17)
- Crosignani, Tittarelli (18)
- Crump (19)
- Glass, Gray (20)
- Glass, Schnatter (21)
- Hayes, Yin (22)
- Raaschou-Nielsen, Hertel (23)
- Richardson (24)
- Rinsky, Hornung (25)
- Schnatter, Glass (26)
- Schnatter, Armstrong (27)
- Stenehjem, Kjærheim (28)
- Talibov, Lehtinen-Jacks (29)
- Vinceti, Rothman (30)

However, most of the identified studies are relatively old (published before the year 2000) and focus almost exclusively on occupational exposure, which in most cases was not measured but estimated by experts based on the workers' activities. The benzene exposure levels in the workplace-related studies are significantly higher than the concentrations measured in indoor air in the GerES V study. Therefore, no risk estimate from the epidemiological studies was used; instead, an established risk estimate from the World Health Organization was applied.

# Bisphenol A

Prior to the search, 13 health outcomes associated with bisphenol A exposure were selected and they formed the basis for the search strings. The search yielded 25,371 hits in total. However, initial checks revealed that the hits included too many studies that were not relevant, such as animal or toxicological studies. Therefore, the search was adjusted and bisphenol A was combined with the search term “epidemiological study”.

**Search string example for Pubmed:**

(((case-control OR (“cohort study” OR cohort-study))) OR ((epidemiology[MeSH Terms] OR epidemiological methods[MeSH Terms] OR epidemiologic measurements[MeSH Terms] OR epidemiology OR epidemiological OR epidemiologic))) AND (bisphenol[Title/Abstract] OR BPA[Title/Abstract])

**BPA specific exclusion criteria:**

- Consideration of the overall group of phenols without differentiation into specific substances such as bisphenol A, B, C, E, F, etc.
- Use of the abbreviation BPA in a different context (e.g., brachial plexus avulsion, balloon pulmonary angioplasty, or the British Paediatric Association)
- Materials studies, for example on the durability and functionality of coatings or dental fillings
- Studies measuring BPA residues in food as well as, for example, in wastewater, sludge, or on rock
- Studies analyzing methods for identifying or removing BPA from (liquid) substances

A total of 6,202 publications were identified through the search. After removal of duplicates, 4,562 were considered potentially eligible for inclusion. Following title and abstract screening, 175 publications remained for full-text assessment. In addition, two further publications were identified through manual searches of reference lists. Of these, a total of 44 full-text articles were deemed relevant to the research question and met the inclusion criteria; these were subsequently subjected to quality assessment. As a result, 40 studies were rated as good or of sufficient methodological quality and were therefore included in the further analysis. Four studies were excluded at a later stage due to insufficient quality. A further nine of the 40 initially included studies had to be excluded. In two studies, BPA was measured not in urine but in blood or serum (31, 32). In two additional studies, total BPA was not assessed; instead, only individual conjugated forms of BPA were measured (33, 34).

Several of the included studies on obesity and diabetes mellitus were based on the same study population from the US study National Health and Nutrition Examination Survey (NHANES). To avoid duplication of data, only those studies were retained for further analysis that were either more recent or were based on a larger portion of the evaluated NHANES dataset. Therefore, five studies were excluded (35-39).

Across the 31 individual studies ultimately included, a total of 18 different health outcomes were investigated in association with BPA. For half of these outcomes, only a single study was identified, which were therefore excluded. Overall, five endpoints were specifically investigated in the group of children and adolescents: obesity (three studies), asthma (two studies), cough (two studies), depression (one study), and attention deficit hyperactivity disorder (ADHD, one study). Bases on the criterion for conducting a meta-analysis - namely, at least three individual studies per endpoint - was met exclusively for the endpoint obesity. Consequently, only this endpoint was taken forward for further evaluation. The studies on obesity used different exposure assessment methods for obesity (continues and categorical scale), therefore a pooling was not possible. The most recent study was thus selected.

**ADHS**

- Children/ adolescents: Tewar, Auinger (40)

**Obesity**

- Children/ adolescents: Eng, Lee (41); Pornkunwilai, Nosoongnoen (42); Wang, Zhou (43)
- Adults: Schulz, Conrad (44), Umweltbundesamt (45); Wang, Li (46)

**Angina pectoris**

- Adults: Melzer, Rice (47)

**Arthritis**

- Adults: Lang, Galloway (48)

**Asthma**

- Children/ adolescents: Donohue, Miller (49); Kim, Kim (50)
- Adults: Lang, Galloway (48); Vaidya and Kulkarni (51)

**Hypertension**

- Adults: Bae, Kim (52); Shankar and Teppala (53); Shiue (54)

**Breast cancer**

- Adults: Morgan, Deoraj (55)

**Chronic Bronchitis**

- Adults: Lang, Galloway (48)

**Depression**

- Children/ adolescents: Perera, Nolte (56)

**Type 1 diabetes mellitus**

- Adults: LaKind, Goodman (57); Ning, Bi (58); Tai and Chen (59)

**Type 2 diabetes mellitus**

- Adults: Ahmadkhaniha, Mansouri (60); Andra, Kalyvas (61); Kim and Park (62); Silver, O'Neill (63); Sun, Cornelis (64)

**Endometriois**

- Adults: Rashidi, Amanlou (65); Louis, Peterson (66); Upson, Sathyanarayana (67)

**Myocardial infarction**

- Adults: LaKind, Goodman (57)

**Cough**

- Children/ adolescents: Donohue, Miller (49); Kim, Kim (50)

**Coronary heart disease**

- Adults: LaKind, Goodman (57); Melzer, Gates (68); Melzer, Osborne (69)

**Polycystic ovary syndrome**

- Adults: Vagi, Azziz-Baumgartner (70)

**Thyroid cancer**

- Adults: Zhou, Zhang (71)

**Stroke**

- Adults: Lang, Galloway (34)

# Cadmium

Before the search, potential health effects of cadmium were explored in various review articles, with a specific focus on children and adolescents in our age group.

**Included**:

- Kidney damage (72)
- Effects on cognitive abilities (e.g., reduced IQ) (72-74)
- Effects on behavior (e.g., ADHD) (74, 75)

**Excluded**:

- Effects on bones (e.g., reduced bone density, increased risk of fractures) (72, 73); reason: effects in children are unlikely
- Lung damage (72); reason: Effects from cadmium inhalation cannot be linked to measured cadmium concentrations in urine (GerES V)
- (Reduced) birth weight in newborns (72); reason: Effects caused by prenatal exposure
- Skeletal anomalies or malformations in newborns (72); reason: association has only been demonstrated in animal studies and effects are caused by prenatal exposure
- Immunosuppressive effects (76); reason: Represents only a preclinical stage and cannot be directly associated with a disease

For the selected health effects, search strings were developed, with searches for cognitive and behavioral effects combined. In addition, a specific search string focusing on the population of children and adolescents was integrated into the query.

**Cadmium specific exclusion criteria:**

- Study population: adults (≥18 years)
- Cadmium concentrations measured in hair, nails, teeth, or any body tissue
- Cadmium examined exclusively as a biomarker in relation to the health effects under consideration

We identified 1,089 publications from which 757 were potentially suitable for inclusion after duplicates were removed. After title and abstract screening, 64 publications remained for full text review. Five additional publications were identified via hand search. Of the total 69 full texts, five were finally classified as relevant for the quantification.

**ADHS**

- Ciesielski, Weuve (77)
- Lee, Jacobs (78)

**Autism spectrum disorder**

- Nabgha-e-Amen, Eqani (79)

**Hearing** **loss**

- Liu, Huo (80)

**Kidney damage, clinically relevant**

- Swaddiwudhipong, Mahasakpan (81)

However, none of these studies reported significant exposure response function. Thus, no risk estimate was selected for cadmium.

# Secondhand smoke

Toxicologists at the German Environment Agency identified 11 health outcomes associated with secondhand smoke (asthma, bronchitis, pneumonia, neurodermatitis, heart disease, cancer, leukemia, lung cancer, otitis media, stroke, sudden infant death syndrome), and search strings were developed accordingly. The literature search focused on existing systematic reviews and meta-analyses, limited to publications from 2012 onward.

**Secondhand smoke specific exclusion criteria:**

- No systematic review or meta-analysis
- Umbrella reviews
- Publications published before 2012
- Tobacco smoke is not separated from other smoke

The time constrain reduced 1,100 de-duplicated hits to 270, of which 37 meta-analyses met selection criteria and 14 additional publications were added from reference screening. 51 publications were quality-assessed using adapted AMSTAR 2 criteria, with 34 rated good or adequate. Meta-analyses were prioritized over systematic reviews, resulting in 32 included for evaluation, 10 of which addressed nine health outcomes for children and adolescents. Due to missing prevalence data for two outcomes in Germany (wheeze and food allergy) and the need for a consistent secondhand smoke exposure definition, only six meta-analyses covering seven outcomes were selected for disease burden estimations listed below. A more comprehensive account of the literature search will be published in a separate publication.

**Asthma**

- Tinuoye, Pell (82)

**Atopic dermatitis**

- Saulyte, Regueira (83), Saulyte, Regueira (84)

**Allergic rhinitis (hey fever)**

- Saulyte, Regueira (83), Saulyte, Regueira (84)

**Otitis media (middle ear infection)**

- Jones, Hassanien (85)

**Lower respiratory tract infections**

- Jayes, Haslam (86)

**Sudden infant death syndrome**

- Zhang and Wang (87)

**Invasive meningococcal disease**

- Murray, Britton (88)

# Reference

1. Chen Y-C, Su H-JJ, Guo Y-LL, Hsueh Y-M, Smith TJ, Ryan LM, et al. Arsenic Methylation and Bladder Cancer Risk in Taiwan. *Cancer Causes & Control* (2003) 14(4):303-10. doi: 10.1023/A:1023905900171.

2. Chung C-J, Huang Y-L, Huang Y-K, Wu M-M, Chen S-Y, Hsueh Y-M, et al. Urinary Arsenic Profiles and the Risks of Cancer Mortality: A Population-Based 20-Year Follow-up Study in Arseniasis-Endemic Areas in Taiwan. *Environmental research* (2013) 122:25-30. doi: <https://doi.org/10.1016/j.envres.2012.11.007>.

3. Huang Y-K, Huang Y-L, Hsueh Y-M, Yang M-H, Wu M-M, Chen S-Y, et al. Arsenic Exposure, Urinary Arsenic Speciation, and the Incidence of Urothelial Carcinoma: A Twelve-Year Follow-up Study. *Cancer Causes & Control* (2008) 19(8):829-39. doi: 10.1007/s10552-008-9146-5.

4. Huang Y-K, Pu Y-S, Chung C-J, Shiue H-S, Yang M-H, Chen C-J, et al. Plasma Folate Level, Urinary Arsenic Methylation Profiles, and Urothelial Carcinoma Susceptibility. *Food and Chemical Toxicology* (2008) 46(3):929-38. doi: <https://doi.org/10.1016/j.fct.2007.10.017>.

5. Melak D, Ferreccio C, Kalman D, Parra R, Acevedo J, Pérez L, et al. Arsenic Methylation and Lung and Bladder Cancer in a Case-Control Study in Northern Chile. *Toxicology and Applied Pharmacology* (2014) 274(2):225-31. doi: <https://doi.org/10.1016/j.taap.2013.11.014>.

6. Pu Y-S, Yang S-M, Huang Y-K, Chung C-J, Huang SK, Chiu AW-H, et al. Urinary Arsenic Profile Affects the Risk of Urothelial Carcinoma Even at Low Arsenic Exposure. *Toxicology and Applied Pharmacology* (2007) 218(2):99-106. doi: <https://doi.org/10.1016/j.taap.2006.09.021>.

7. Steinmaus C, Bates MN, Yuan Y, Kalman D, Atallah R, Rey OA, et al. Arsenic Methylation and Bladder Cancer Risk in Case-Control Studies in Argentina and the United States. *Journal of Occupational and Environmental Medicine* (2006) 48(5):478-88.

8. Wu C-C, Chen M-C, Huang Y-K, Huang C-Y, Lai L-A, Chung C-J, et al. Environmental Tobacco Smoke and Arsenic Methylation Capacity Are Associated with Urothelial Carcinoma. *Journal of the Formosan Medical Association* (2013) 112(9):554-60. doi: <https://doi.org/10.1016/j.jfma.2013.05.012>.

9. Argos M, Parvez F, Rahman M, Rakibuz-Zaman M, Ahmed A, Hore SK, et al. Arsenic and Lung Disease Mortality in Bangladeshi Adults. *Epidemiology* (2014) 25(4):536-43. doi: 10.1097/ede.0000000000000106.

10. García-Esquinas E, Pollán M, Umans JG, Francesconi KA, Goessler W, Guallar E, et al. Arsenic Exposure and Cancer Mortality in a Us-Based Prospective Cohort: The Strong Heart Study. *Cancer Epidemiology, Biomarkers & Prevention* (2013) 22(11):1944-53. doi: 10.1158/1055-9965.Epi-13-0234-t.

11. Steinmaus C, Yuan Y, Kalman D, Rey OA, Skibola CF, Dauphine D, et al. Individual Differences in Arsenic Metabolism and Lung Cancer in a Case-Control Study in Cordoba, Argentina. *Toxicology and Applied Pharmacology* (2010) 247(2):138-45. doi: <https://doi.org/10.1016/j.taap.2010.06.006>.

12. Chen Y-C, Guo Y-LL, Su H-JJ, Hsueh Y-M, Smith TJ, Ryan LM, et al. Arsenic Methylation and Skin Cancer Risk in Southwestern Taiwan. *Journal of Occupational and Environmental Medicine* (2003) 45(3):241-8. doi: 10.1097/01.jom.0000058336.05741.e8.

13. Gilbert-Diamond D, Li Z, Perry AE, Spencer SK, Gandolfi AJ, Karagas MR. A Population-Based Case-Control Study of Urinary Arsenic Species and Squamous Cell Carcinoma in New Hampshire, USA. *Environmental Health Perspectives* (2013) 121(10):1154-60. doi: <http://dx.doi.org/10.1289/ehp.1206178>.

14. Yu RC, Hsu K-H, Chen C-J, Froines JR. Arsenic Methylation Capacity and Skin Cancer. *Cancer Epidemiology, Biomarkers & Prevention* (2000) 9(11):1259-62.

15. Leonardi G, Vahter M, Clemens F, Goessler W, Gurzau E, Hemminki K, et al. Inorganic Arsenic and Basal Cell Carcinoma in Areas of Hungary, Romania, and Slovakia: A Case-Control Study. *Environmental Health Perspectives* (2012) 120(5):721-6. doi: 10.1289/ehp.1103534.

16. Axelsson G, Barregard L, Holmberg E, Sallsten G. Cancer Incidence in a Petrochemical Industry Area in Sweden. *Sci Total Environ* (2010) 408(20):4482-7. doi: 10.1016/j.scitotenv.2010.06.028.

17. Collins JJ, Anteau SE, Swaen GM, Bodner KM, Bodnar CM. Lymphatic and Hematopoietic Cancers among Benzene-Exposed Workers. *J Occup Environ Med* (2015) 57(2):159-63. doi: 10.1097/jom.0000000000000324.

18. Crosignani P, Tittarelli A, Borgini A, Codazzi T, Rovelli A, Porro E, et al. Childhood Leukemia and Road Traffic: A Population-Based Case-Control Study. *Int J Cancer* (2004) 108(4):596-9. doi: 10.1002/ijc.11597.

19. Crump KS. Risk of Benzene-Induced Leukemia: A Sensitivity Analysis of the Pliofilm Cohort with Additional Follow-up and New Exposure Estimates. *J Toxicol Environ Health* (1994) 42(2):219-42. doi: 10.1080/15287399409531875.

20. Glass DC, Gray CN, Jolley DJ, Gibbons C, Sim MR, Fritschi L, et al. Leukemia Risk Associated with Low-Level Benzene Exposure. *Epidemiology* (2003) 14(5):569-77. doi: 10.1097/01.ede.0000082001.05563.e0.

21. Glass DC, Schnatter AR, Tang G, Irons RD, Rushton L. Risk of Myeloproliferative Disease and Chronic Myeloid Leukaemia Following Exposure to Low-Level Benzene in a Nested Case-Control Study of Petroleum Workers. *Occup Environ Med* (2014) 71(4):266-74. Epub 20140214. doi: 10.1136/oemed-2013-101664.

22. Hayes RB, Yin SN, Dosemeci M, Li GL, Wacholder S, Travis LB, et al. Benzene and the Dose-Related Incidence of Hematologic Neoplasms in China. Chinese Academy of Preventive Medicine--National Cancer Institute Benzene Study Group. *J Natl Cancer Inst* (1997) 89(14):1065-71. doi: 10.1093/jnci/89.14.1065.

23. Raaschou-Nielsen O, Hertel O, Thomsen BL, Olsen JH. Air Pollution from Traffic at the Residence of Children with Cancer. *Am J Epidemiol* (2001) 153(5):433-43. doi: 10.1093/aje/153.5.433.

24. Richardson DB. Temporal Variation in the Association between Benzene and Leukemia Mortality. *Environ Health Perspect* (2008) 116(3):370-4. doi: 10.1289/ehp.10841.

25. Rinsky RA, Hornung RW, Silver SR, Tseng CY. Benzene Exposure and Hematopoietic Mortality: A Long-Term Epidemiologic Risk Assessment. *Am J Ind Med* (2002) 42(6):474-80. doi: 10.1002/ajim.10138.

26. Schnatter AR, Glass DC, Tang G, Irons RD, Rushton L. Myelodysplastic Syndrome and Benzene Exposure among Petroleum Workers: An International Pooled Analysis. *J Natl Cancer Inst* (2012) 104(22):1724-37. Epub 20121030. doi: 10.1093/jnci/djs411.

27. Schnatter AR, Armstrong TW, Thompson LS, Nicolich MJ, Katz AM, Huebner WW, et al. The Relationship between Low-Level Benzene Exposure and Leukemia in Canadian Petroleum Distribution Workers. *Environ Health Perspect* (1996) 104 Suppl 6(Suppl 6):1375-9. doi: 10.1289/ehp.961041375.

28. Stenehjem JS, Kjærheim K, Bråtveit M, Samuelsen SO, Barone-Adesi F, Rothman N, et al. Benzene Exposure and Risk of Lymphohaematopoietic Cancers in 25 000 Offshore Oil Industry Workers. *Br J Cancer* (2015) 112(9):1603-12. Epub 20150324. doi: 10.1038/bjc.2015.108.

29. Talibov M, Lehtinen-Jacks S, Martinsen JI, Kjærheim K, Lynge E, Sparén P, et al. Occupational Exposure to Solvents and Acute Myeloid Leukemia: A Population-Based, Case-Control Study in Four Nordic Countries. *Scand J Work Environ Health* (2014) 40(5):511-7. Epub 20140519. doi: 10.5271/sjweh.3436.

30. Vinceti M, Rothman KJ, Crespi CM, Sterni A, Cherubini A, Guerra L, et al. Leukemia Risk in Children Exposed to Benzene and Pm10 from Vehicular Traffic: A Case-Control Study in an Italian Population. *Eur J Epidemiol* (2012) 27(10):781-90. Epub 20120815. doi: 10.1007/s10654-012-9727-1.

31. Aekplakorn W, Chailurkit LO, Ongphiphadhanakul B. Relationship of Serum Bisphenol a with Diabetes in the Thai Population, National Health Examination Survey Iv, 2009. *Journal of diabetes* (2015) 7(2):240-9. Epub 2014/04/12. doi: 10.1111/1753-0407.12159.

32. Akin L, Kendirci M, Narin F, Kurtoglu S, Saraymen R, Kondolot M, et al. The Endocrine Disruptor Bisphenol a May Play a Role in the Aetiopathogenesis of Polycystic Ovary Syndrome in Adolescent Girls. *Acta paediatrica (Oslo, Norway : 1992)* (2015) 104(4):e171-7. Epub 2014/12/04. doi: 10.1111/apa.12885.

33. Trabert B, Falk RT, Figueroa JD, Graubard BI, Garcia-Closas M, Lissowska J, et al. Urinary Bisphenol a-Glucuronide and Postmenopausal Breast Cancer in Poland. *Cancer causes & control : CCC* (2014) 25(12):1587-93. Epub 2014/09/06. doi: 10.1007/s10552-014-0461-8.

34. Wang IJ, Chen CY, Bornehag CG. Bisphenol a Exposure May Increase the Risk of Development of Atopic Disorders in Children. *Int J Hyg Environ Health* (2016) 219(3):311-6. Epub 2016/01/15. doi: 10.1016/j.ijheh.2015.12.001.

35. Bhandari R, Xiao J, Shankar A. Urinary Bisphenol a and Obesity in U.S. Children. *American journal of epidemiology* (2013) 177(11):1263-70. Epub 2013/04/06. doi: 10.1093/aje/kws391.

36. Carwile JL, Michels KB. Urinary Bisphenol a and Obesity: Nhanes 2003-2006. *Environmental research* (2011) 111(6):825-30. Epub 2011/06/17. doi: 10.1016/j.envres.2011.05.014.

37. Casey MF, Neidell M. Disconcordance in Statistical Models of Bisphenol a and Chronic Disease Outcomes in Nhanes 2003-08. *PLoS ONE* (2013) 8(11):e79944. Epub 2013/11/14. doi: 10.1371/journal.pone.0079944.

38. Shankar A, Teppala S. Relationship between Urinary Bisphenol a Levels and Diabetes Mellitus. *The Journal of clinical endocrinology and metabolism* (2011) 96(12):3822-6. Epub 2011/10/01. doi: 10.1210/jc.2011-1682.

39. Trasande L, Attina TM, Blustein J. Association between Urinary Bisphenol a Concentration and Obesity Prevalence in Children and Adolescents. *Jama* (2012) 308(11):1113-21. Epub 2012/09/20. doi: 10.1001/2012.jama.11461.

40. Tewar S, Auinger P, Braun JM, Lanphear B, Yolton K, Epstein JN, et al. Association of Bisphenol a Exposure and Attention-Deficit/Hyperactivity Disorder in a National Sample of U.S. Children. *Environmental research* (2016) 150:112-8. Epub 2016/06/10. doi: 10.1016/j.envres.2016.05.040.

41. Eng DS, Lee JM, Gebremariam A, Meeker JD, Peterson K, Padmanabhan V. Bisphenol a and Chronic Disease Risk Factors in Us Children. *Pediatrics* (2013) 132(3):e637-45. Epub 2013/08/21. doi: <https://doi.org/10.1542/peds.2013-0106>.

42. Pornkunwilai S, Nosoongnoen W, Jantarat C, Wachrasindhu S, Supornsilchai V. Urinary Bisphenol a Detection Is Significantly Associated with Young and Obese Thai Children. *Asian Biomedicine* (2015) 9(3):363-72. doi: 10.5372/1905-7415.0903.405.

43. Wang H-x, Zhou Y, Tang C-x, Wu J-g, Chen Y, Jiang Q-w. Association between Bisphenol a Exposure and Body Mass Index in Chinese School Children a Cross-Sectional Study. *Environmental Health* (2012) 11(1).

44. Schulz C, Conrad A, Becker K, Kolossa-Gehring M, Seiwert M, Seifert B. Twenty Years of the German Environmental Survey (Geres): Human Biomonitoring – Temporal and Spatial (West Germany/East Germany) Differences in Population Exposure. *International Journal of Hygiene and Environmental Health* (2007) 210(3):271-97. doi: <https://doi.org/10.1016/j.ijheh.2007.01.034>.

45. Umweltbundesamt. Deutsche Umweltstudie Zur Gesundheit, Geres Vi (2023-2024). (2022) [11. Oktober 2022]. Available from: <https://www.umweltbundesamt.de/themen/gesundheit/belastung-des-menschen-ermitteln/deutsche-umweltstudie-zur-gesundheit-geres/deutsche-umweltstudie-zur-gesundheit-geres-vi-2023>.

46. Wang T, Li M, Chen B, Xu M, Xu Y, Huang Y, et al. Urinary Bisphenol a (Bpa) Concentration Associates with Obesity and Insulin Resistance. *The Journal of clinical endocrinology and metabolism* (2012) 97(2):E223-7. Epub 2011/11/18. doi: 10.1210/jc.2011-1989.

47. Melzer D, Rice NE, Lewis C, Henley WE, Galloway TS. Association of Urinary Bisphenol a Concentration with Heart Disease: Evidence from Nhanes 2003/06. *PLoS ONE* (2010) 5(1):e8673. Epub 2010/01/20. doi: 10.1371/journal.pone.0008673.

48. Lang IA, Galloway TS, Scarlett A, Henley WE, Depledge M, Wallace RB, et al. Association of Urinary Bisphenol a Concentration with Medical Disorders and Laboratory Abnormalities in Adults. *Jama* (2008) 300(11):1303-10. Epub 2008/09/19. doi: 10.1001/jama.300.11.1303.

49. Donohue KM, Miller RL, Perzanowski MS, Just AC, Hoepner LA, Arunajadai S, et al. Prenatal and Postnatal Bisphenol a Exposure and Asthma Development among Inner-City Children. *The Journal of allergy and clinical immunology* (2013) 131(3):736-42. Epub 2013/03/05. doi: 10.1016/j.jaci.2012.12.1573.

50. Kim KN, Kim JH, Kwon HJ, Hong SJ, Kim BJ, Lee SY, et al. Bisphenol a Exposure and Asthma Development in School-Age Children: A Longitudinal Study. *PLoS ONE* (2014) 9(10):e111383. Epub 2014/10/31. doi: 10.1371/journal.pone.0111383.

51. Vaidya SV, Kulkarni H. Association of Urinary Bisphenol a Concentration with Allergic Asthma: Results from the National Health and Nutrition Examination Survey 2005-2006. *The Journal of asthma : official journal of the Association for the Care of Asthma* (2012) 49(8):800-6. Epub 2012/09/11. doi: 10.3109/02770903.2012.721041.

52. Bae S, Kim JH, Lim YH, Park HY, Hong YC. Associations of Bisphenol a Exposure with Heart Rate Variability and Blood Pressure. *Hypertension (Dallas, Tex : 1979)* (2012) 60(3):786-93. Epub 2012/08/02. doi: 10.1161/hypertensionaha.112.197715.

53. Shankar A, Teppala S. Urinary Bisphenol a and Hypertension in a Multiethnic Sample of Us Adults. *Journal of environmental and public health* (2012) 2012:481641. Epub 2012/03/01. doi: 10.1155/2012/481641.

54. Shiue I. Higher Urinary Heavy Metal, Arsenic, and Phthalate Concentrations in People with High Blood Pressure: Us Nhanes, 2009-2010. *Blood pressure* (2014) 23(6):363-9. Epub 2014/06/20. doi: 10.3109/08037051.2014.925228.

55. Morgan M, Deoraj A, Felty Q, Roy D. Environmental Estrogen-Like Endocrine Disrupting Chemicals and Breast Cancer. *Molecular and cellular endocrinology* (2017) 457:89-102. Epub 2016/10/19. doi: 10.1016/j.mce.2016.10.003.

56. Perera F, Nolte ELR, Wang Y, Margolis AE, Calafat AM, Wang S, et al. Bisphenol a Exposure and Symptoms of Anxiety and Depression among Inner City Children at 10–12 Years of Age. *Environmental research* (2016) 151:195-202. doi: 10.1016/j.envres.2016.07.028.

57. LaKind JS, Goodman M, Naiman DQ. Use of Nhanes Data to Link Chemical Exposures to Chronic Diseases: A Cautionary Tale. *PLoS ONE* (2012) 7(12):e51086. Epub 2012/12/12. doi: 10.1371/journal.pone.0051086.

58. Ning G, Bi Y, Wang T, Xu M, Xu Y, Huang Y, et al. Relationship of Urinary Bisphenol a Concentration to Risk for Prevalent Type 2 Diabetes in Chinese Adults: A Cross-Sectional Analysis. *Annals of internal medicine* (2011) 155(6):368-74. Epub 2011/09/21. doi: 10.7326/0003-4819-155-6-201109200-00005.

59. Tai X, Chen Y. Urinary Bisphenol a Concentrations Positively Associated with Glycated Hemoglobin and Other Indicators of Diabetes in Canadian Men. *Environmental research* (2016) 147:172-8. Epub 2016/02/19. doi: 10.1016/j.envres.2016.02.006.

60. Ahmadkhaniha R, Mansouri M, Yunesian M, Omidfar K, Jeddi MZ, Larijani B, et al. Association of Urinary Bisphenol a Concentration with Type-2 Diabetes Mellitus. *Journal of environmental health science & engineering* (2014) 12(1):64. Epub 2014/03/15. doi: 10.1186/2052-336x-12-64.

61. Andra SS, Kalyvas H, Andrianou XD, Charisiadis P, Christophi CA, Makris KC. Preliminary Evidence of the Association between Monochlorinated Bisphenol a Exposure and Type Ii Diabetes Mellitus: A Pilot Study. *Journal of environmental science and health Part A, Toxic/hazardous substances & environmental engineering* (2015) 50(3):243-59. Epub 2015/01/17. doi: 10.1080/10934529.2015.981111.

62. Kim K, Park H. Association between Urinary Concentrations of Bisphenol a and Type 2 Diabetes in Korean Adults: A Population-Based Cross-Sectional Study. *Int J Hyg Environ Health* (2013) 216(4):467-71. Epub 2012/08/28. doi: 10.1016/j.ijheh.2012.07.007.

63. Silver M, O'Neill M, Sowers M, Park S. Urinary Bisphenol a and Type-2 Diabetes in U.S. Adults Data from Nhanes 2003-2008. *PLoS ONE, Vol 6, Iss 10, p e* (2011) 26868.

64. Sun Q, Cornelis MC, Townsend MK, Tobias DK, Heather Eliassen A, Franke AA, et al. Association of Urinary Concentrations of Bisphenol a and Phthalate Metabolites with Risk of Type 2 Diabetes: A Prospective Investigation in the Nurses' Health Study (Nhs) and Nhsii Cohorts. *Environmental Health Perspectives* (2014) 122(6):616-23. doi: 10.1289/ehp.1307201.

65. Rashidi BH, Amanlou M, Lak TB, Ghazizadeh M, Eslami B. A Case-Control Study of Bisphenol a and Endometrioma among Subgroup of Iranian Women. *Journal of research in medical sciences : the official journal of Isfahan University of Medical Sciences* (2017) 22:7. Epub 2017/04/13. doi: 10.4103/1735-1995.199086.

66. Louis GMB, Peterson CM, Chen Z, Croughan M, Sundaram R, Stanford J, et al. Bisphenol a and Phthalates and Endometriosis: The Endometriosis: Natural History, Diagnosis and Outcomes Study. *Fertility and sterility* (2013) 100(1):162-9.e1-2. Epub 2013/04/13. doi: 10.1016/j.fertnstert.2013.03.026.

67. Upson K, Sathyanarayana S, De Roos AJ, Koch HM, Scholes D, Holt VL. A Population-Based Case-Control Study of Urinary Bisphenol a Concentrations and Risk of Endometriosis. *Human reproduction (Oxford, England)* (2014) 29(11):2457-64. Epub 2014/09/11. doi: 10.1093/humrep/deu227.

68. Melzer D, Gates P, Osborne NJ, Henley WE, Cipelli R, Young A, et al. Urinary Bisphenol a Concentration and Angiography-Defined Coronary Artery Stenosis. *PLoS ONE, Vol 7, Iss 8, p e* (2012) 43378.

69. Melzer D, Osborne NJ, Henley WE, Cipelli R, Young A, Money C, et al. Urinary Bisphenol a Concentration and Risk of Future Coronary Artery Disease in Apparently Healthy Men and Women. *Circulation* (2012) 125(12):1482-90. Epub 2012/02/23. doi: 10.1161/circulationaha.111.069153.

70. Vagi SJ, Azziz-Baumgartner E, Sjödin A, Calafat AM, Dumesic D, Gonzalez L, et al. Exploring the Potential Association between Brominated Diphenyl Ethers, Polychlorinated Biphenyls, Organochlorine Pesticides, Perfluorinated Compounds, Phthalates, and Bisphenol a in Polycystic Ovary Syndrome: A Case-Control Study. *BMC Endocrine Disorders* (2014) 14(1). doi: 10.1186/1472-6823-14-86.

71. Zhou Z, Zhang J, Jiang F, Xie Y, Zhang X, Jiang L. Higher Urinary Bisphenol a Concentration and Excessive Iodine Intake Are Associated with Nodular Goiter and Papillary Thyroid Carcinoma. *Bioscience Reports* (2017) 37(4). doi: 10.1042/BSR20170678.

72. Agency for Toxic Substances and Disease Registry. Toxicological Profile for Cadmium. Atlanta, GA, USA: (2012).

73. Lamkarkach F, Ougier E, Garnier R, Viau C, Kolossa-Gehring M, Lange R, et al. Human Biomonitoring Initiative (Hbm4eu): Human Biomonitoring Guidance Values (Hbm-Gvs) Derived for Cadmium and Its Compounds. *Environment international* (2021) 147:106337. doi: <https://doi.org/10.1016/j.envint.2020.106337>.

74. Sanders AP, Claus Henn B, Wright RO. Perinatal and Childhood Exposure to Cadmium, Manganese, and Metal Mixtures and Effects on Cognition and Behavior: A Review of Recent Literature. *Current Environmental Health Reports* (2015) 2(3):284-94. doi: <https://doi.org/10.1007/s40572-015-0058-8>.

75. Schoeters G, Den Hond E, Zuurbier M, Naginiene R, van den Hazel P, Stilianakis N, et al. Cadmium and Children: Exposure and Health Effects. *Acta Paediatr Suppl* (2006) 95(453):50-4. doi: <https://doi.org/10.1080/08035320600886232>.

76. Schoeters G, Den Hond E, Zuurbier M, Naginiene R, van den Hazel P, Stilianakis N, et al. Cadmium and Children: Exposure and Health Effects. (2006) 95(453):50-4. doi: 10.1080/08035320600886232.

77. Ciesielski T, Weuve J, Bellinger DC, Schwartz J, Lanphear B, Wright RO. Cadmium Exposure and Neurodevelopmental Outcomes in U.S. Children. *Environmental Health Perspectives* (2012) 120(5):758-63. doi: doi:10.1289/ehp.1104152.

78. Lee D-H, Jacobs DR, Porta M. Association of Serum Concentrations of Persistent Organic Pollutants with the Prevalence of Learning Disability and Attention Deficit Disorder. *Journal of Epidemiology and Community Health* (2007) 61(7):591. doi: 10.1136/jech.2006.054700.

79. Nabgha-e-Amen, Eqani SAMAS, Khuram F, Alamdar A, Tahir A, Shah STA, et al. Environmental Exposure Pathway Analysis of Trace Elements and Autism Risk in Pakistani Children Population. *Science of The Total Environment* (2020) 712:136471. doi: <https://doi.org/10.1016/j.scitotenv.2019.136471>.

80. Liu Y, Huo X, Xu L, Wei X, Wu W, Wu X, et al. Hearing Loss in Children with E-Waste Lead and Cadmium Exposure. *Science of The Total Environment* (2018) 624:621-7. doi: <https://doi.org/10.1016/j.scitotenv.2017.12.091>.

81. Swaddiwudhipong W, Mahasakpan P, Jeekeeree W, Funkhiew T, Sanjum R, Apiwatpaiboon T, et al. Renal and Blood Pressure Effects from Environmental Cadmium Exposure in Thai Children. *Environmental research* (2015) 136:82-7. doi: <https://doi.org/10.1016/j.envres.2014.10.017>.

82. Tinuoye O, Pell JP, Mackay DF. Meta-Analysis of the Association between Secondhand Smoke Exposure and Physician-Diagnosed Childhood Asthma. *Nicotine & Tobacco Research* (2013) 15(9):1475-83. doi: 10.1093/ntr/ntt033.

83. Saulyte J, Regueira C, Montes-Martínez A, Khudyakov P, Takkouche B. Correction: Active or Passive Exposure to Tobacco Smoking and Allergic Rhinitis, Allergic Dermatitis, and Food Allergy in Adults and Children: A Systematic Review and Meta-Analysis. *PLOS Medicine* (2016) 13(2):e1001939. doi: 10.1371/journal.pmed.1001939.

84. Saulyte J, Regueira C, Montes-Martínez A, Khudyakov P, Takkouche B. Active or Passive Exposure to Tobacco Smoking and Allergic Rhinitis, Allergic Dermatitis, and Food Allergy in Adults and Children: A Systematic Review and Meta-Analysis. *PLOS Medicine* (2014) 11(3):e1001611. doi: 10.1371/journal.pmed.1001611.

85. Jones LL, Hassanien A, Cook DG, Britton J, Leonardi-Bee J. Parental Smoking and the Risk of Middle Ear Disease in Children: A Systematic Review and Meta-Analysis. *Archives of Pediatrics & Adolescent Medicine* (2012) 166(1):18-27. doi: 10.1001/archpediatrics.2011.158.

86. Jayes L, Haslam PL, Gratziou CG, Powell P, Britton J, Vardavas C, et al. Smokehaz: Systematic Reviews and Meta-Analyses of the Effects of Smoking on Respiratory Health. *Chest* (2016) 150(1):164-79. doi: <https://doi.org/10.1016/j.chest.2016.03.060>.

87. Zhang K, Wang X. Maternal Smoking and Increased Risk of Sudden Infant Death Syndrome: A Meta-Analysis. *Legal Medicine* (2013) 15(3):115-21. doi: <https://doi.org/10.1016/j.legalmed.2012.10.007>.

88. Murray RL, Britton J, Leonardi-Bee J. Second Hand Smoke Exposure and the Risk of Invasive Meningococcal Disease in Children: Systematic Review and Meta-Analysis. *BMC public health* (2012) 12(1):1062. doi: 10.1186/1471-2458-12-1062.
